# Supplementary material for: Epigenetic interaction between UTX and DNMT1 regulates diet-induced myogenic remodeling in brown fat
Source: Nat Commun. 2021 Nov 25;12:6838. doi: 10.1038/s41467-021-27141-7 (PMC8617140; doi:10.1038/s41467-021-27141-7)
Supplement: Supplementary file 1 — Supplementary Information [file 41467_2021_27141_MOESM1_ESM.pdf]

Supplemental Tables and Figures
Epigenetic Interaction between UTX and DNMT1 Regulates Diet-Induced Myogenic Remodeling in Brown Fat

Fenfen Li[1], Jia Jing[1], Miranda Movahed[1], Xin Cui[1], Qiang Cao[1], Rui Wu[1], Ziyue Chen[2], Liqing Yu[3], Yi Pan[2], Huidong Shi[4,5], Hang Shi[1*], Bingzhong Xue[1*]

[1]Department of Biology, Georgia State University, Atlanta, GA 30303, USA.
[2]Department of Computer Science, Georgia State University, Atlanta, GA 30303, USA
[3]Division of Endocrinology, Diabetes and Nutrition, Department of Medicine, University of Maryland School of Medicine, Baltimore, MD 21201, USA.
[4]Georgia Cancer Center, and [5]Department of Biochemistry and Molecular Biology, Medical College of Georgia, Augusta University, Augusta, GA 30912, USA.

Running title: UTX and DNMT1 regulate myogenic remodeling in BAT.

* Correspondence should be addressed to:
Hang Shi, Department of Biology and Center for Obesity Reversal, Georgia State University, Atlanta, GA 30303, USA. Contact: 404-413-5799, hshi3@gsu.edu.

Bingzhong Xue, Department of Biology and Center for Obesity Reversal, Georgia State University, Atlanta, GA 30303, USA. Contact: 404-413-5747, bxue@gsu.edu

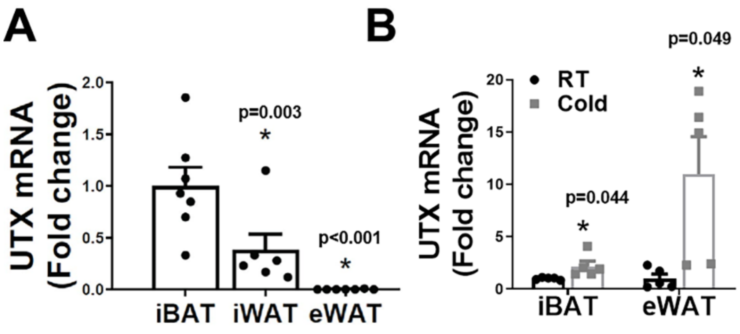

Supplemental figure 1. (**A**) *Utx* mRNA level in interscapular BAT (iBAT), inguinal WAT (iWAT) and epididymal WAT (eWAT) depots of 2-month-old male mice (n=7/group). *indicates statistical significance vs. iBAT by One-Way ANOVA followed by Fisher's Least Significant Difference (LSD) test, F(2,18)=15.17, p<0.0001.
(**B**) *Utx* mRNA level in iBAT and eWAT depots of 2-month-old male mice housed at room temperature (RT) or challenged with a 7-day 5°C cold exposure (n=5/group). *indicates statistical significance between room RT and cold exposure by two-tailed unpaired Student's t-test.
All data are expressed as mean ± SEM.

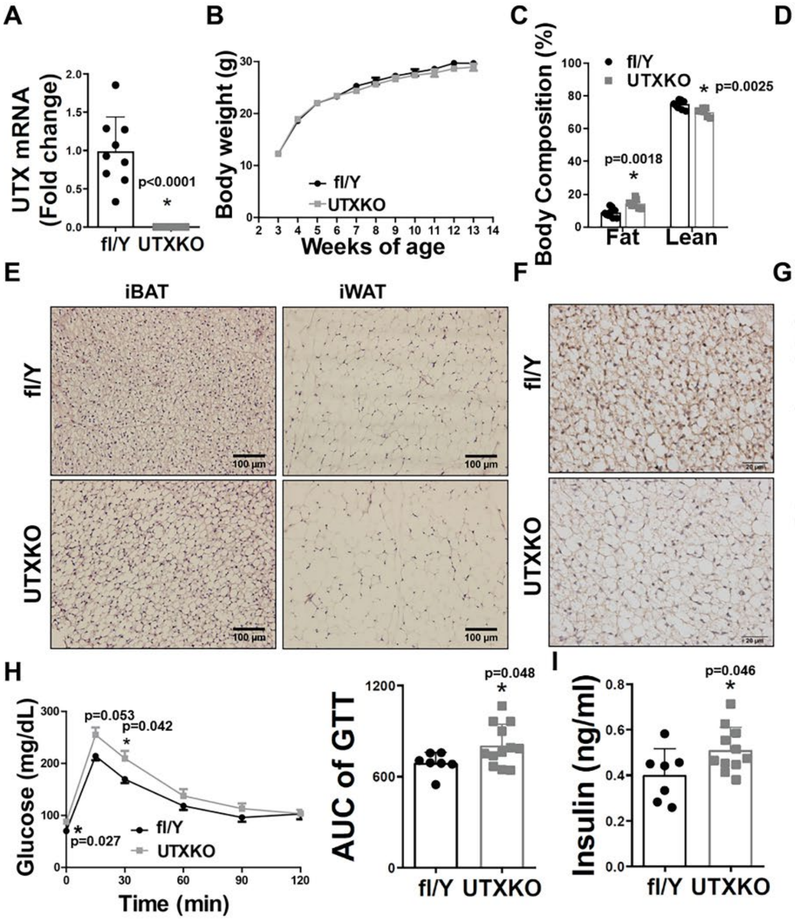

Supplemental figure 2. UTX deficiency in brown fat promotes adiposity in male mice on a chow diet. Male UTXKO and their fl/Y littermates were weaned onto regular chow diet.

(**A**) *Utx* mRNA level in iBAT of male UTXKO and fl/Y mice (n=9/group).

(**B**) Body weight growth curve of male UTXKO mice and fl/Y mice on regular chow diet (n=7 for fl/Y and 11 for UTXKO).

(**C**)-(**D**) Body composition (**C**, n=9 for fl/Y, and 7 for UTXKO), and Fat pad weight (iBAT, iWAT, eWAT and retroperitoneal WAT (rWAT)) (**D**, n=6 for fl/Y and 8 for UTXKO) in male UTXKO and fl/Y mice on regular chow diet.

(**E**)-(**F**) Representative H&E staining of iBAT and iWAT (**E**), and UCP1 immunohistochemistry (IHC) staining in iBAT (**F**) in male UTXKO and fl/Y mice on regular chow diet (n=3 replicates for each group).

(**G**)-(**I**) Fed and fasted circulating glucose (n=7 for fl/Y, 10 for UTXKO) and insulin (n=9 for fl/Y, 10 for UTXKO) levels (**G**), Glucose tolerance test (GTT) (**H**, n=7 for fl/Y, 12 for UTXKO), and Insulin levels at 15 minutes during GTT test (**I**, n=7 for fl/Y, 11 for UTXKO) in male UTXKO and fl/Y mice on regular chow diet.

All data are expressed as mean ± SEM. *indicates statistical significance as marked in each panel between UTXKO and fl/Y by two-tailed unpaired Student's t-test.

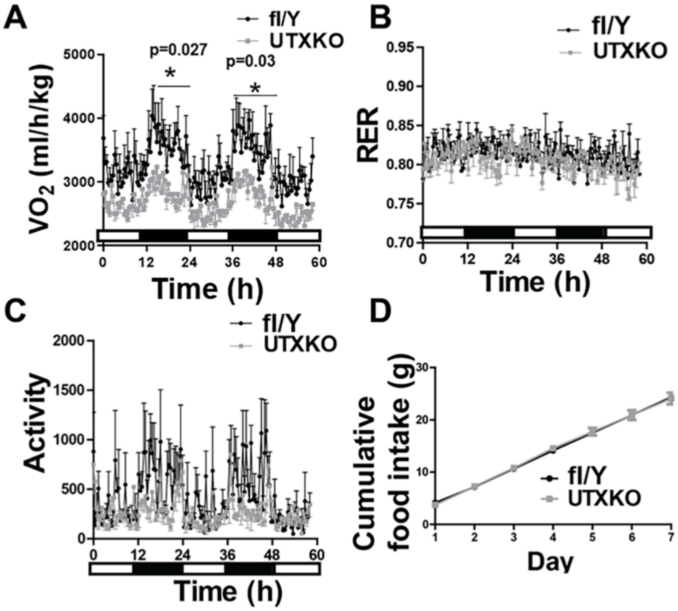

Supplemental figure 3. Metabolic characterization of male UTXKO and fl/Y control mice on HFD. Male UTXKO and their littermate control fl/Y mice were put on HFD when they were 5 weeks of age.

(**A**)-(**D**) Oxygen consumption (VO_2) (**A**), Respiratory exchange rate (RER) (**B**), Locomotor activity (**C**), and Cumulative food intake (**D**) in male UTXKO and fl/Y mice on HFD. For **A**-**C**, n=4/group; for **D**, n=7 for fl/Y and 5 for UTXKO. In (**A**), *indicates statistical significance between fl/fl and D1KO analyzed by ANOVA with repeated measures. Time 17-24 hours, $F(1,6)=8.518$, $p=0.027$; time 36-48 hours, $F(1,6)=8.058$, $p=0.03$.

All data are expressed as mean ± SEM.

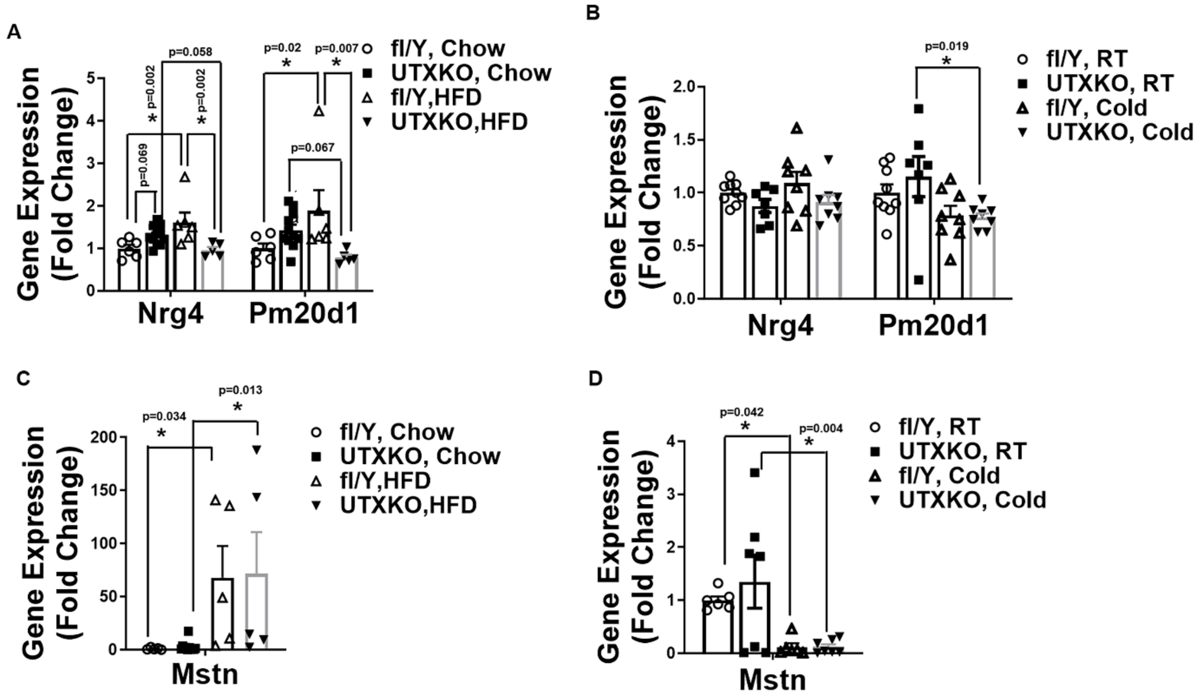

Supplemental Figure 4. Gene expression analysis in iBAT of male UTXKO and fl/Y littermate control mice.

(**A**)-(**B**) *Nrg4* and *Pm20d1* expression in iBAT of male UTXKO and fl/Y littermate control mice fed chow or HFD (**A**, fl/Y chow=6, UTXKO chow=11, fl/Y HFD=6, UTXKO HFD=5) or challenged with cold (**B**, fl/Y RT=9, UTXKO RT=7, fl/Y Cold=8, UTXKO Cold=8). *indicates statistical significance between groups with one-way ANOVA followed by Fisher’s LSD multiple comparisons test: $F(3,24)=5.413$, $p=0.005$ for *Nrg4* in (**A**), $F(3,24)=3.559$, $p=0.029$ for *Pm20d1* in (**A**); $F(3,28)=1.820$, $p=0.166$ for *Nrg4* in (**B**), $F(3,28)=2.849$, $p=0.055$ for *pm20d1* in (**B**).

(**C**)-(**D**) *Mstn* expression in iBAT of male UTXKO and fl/Y littermate control mice fed chow or HFD (**C**, fl/Y chow=5, UTXKO chow=11, fl/Y HFD=5, UTXKO HFD=5) or challenged with cold (**D**, fl/Y RT=6, UTXKO RT=7, fl/Y Cold=6, UTXKO Cold=7). *indicates statistical significance between groups with Kruskal-Wallis non-parametric ANOVA H test by rank followed by Pairwise Comparisons test between groups, $H(3)=15.378$, $p=0.002$ in (**C**), and $H(3)=9.195$, $p=0.027$ in (**D**).

All data are expressed as mean ± SEM.

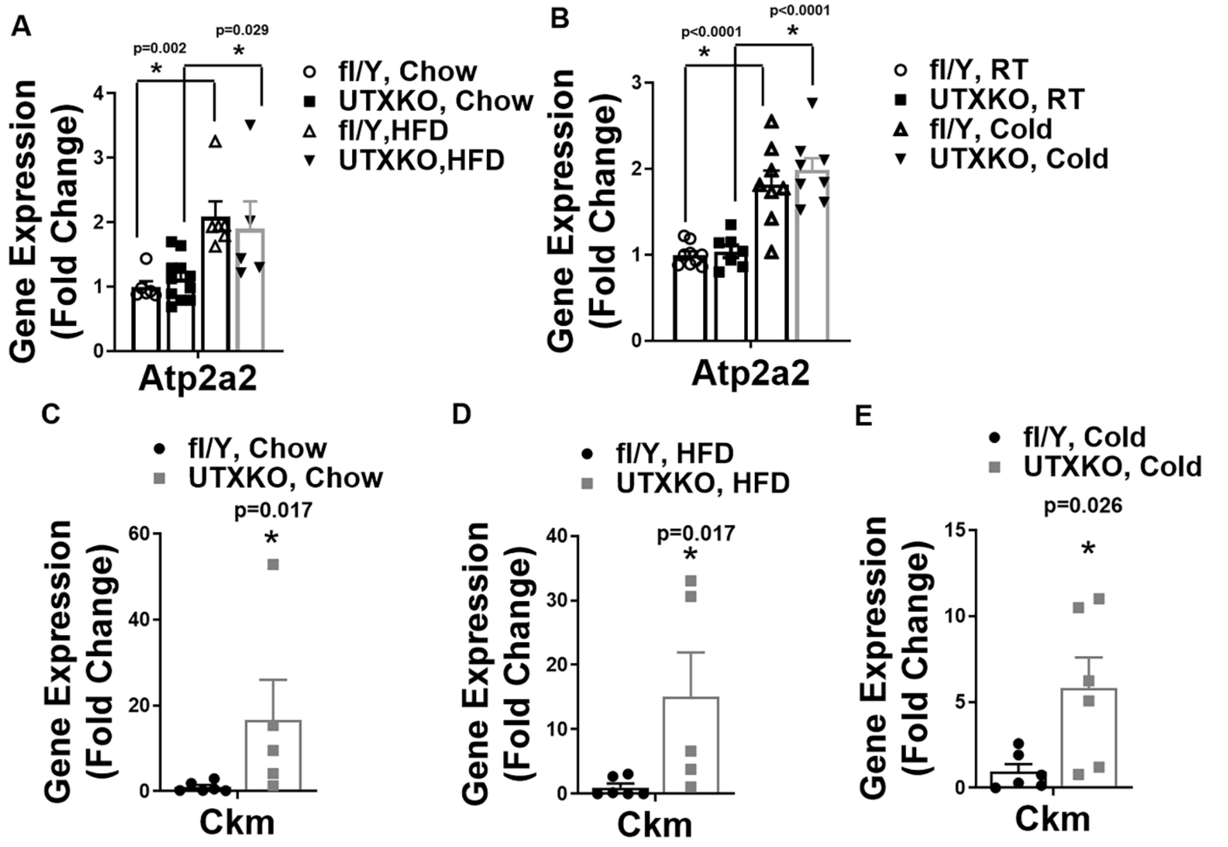

Supplemental Figure 5. Gene expression analysis in iBAT of male UTXKO and fl/Y littermate control mice.

(**A**)-(**B**) *Atp2a2* expression in iBAT of male UTXKO and fl/Y littermate control mice fed chow or HFD (**A**, fl/Y chow=6, UTXKO chow=11, fl/Y HFD=6, UTXKO HFD=5) or challenged with cold (**B**, fl/Y RT=9, UTXKO RT=7, fl/Y Cold=8, UTXKO Cold=8). In (**A**), *indicates statistical significance between groups with Kruskal-Wallis non-parametric ANOVA H test by rank followed by Pairwise Comparisons test between groups, $H(3)=15.165$, $p=0.002$. In (**B**), *indicates statistical significance between groups with one-way ANOVA followed by Fisher's LSD multiple comparisons test, $F(3,28)=20.205$, $p<0.001$.

(**C**)-(**E**) *Ckm* expression in iBAT of male UTXKO and fl/Y littermate control mice fed chow (**C**, fl/Y chow=6, UTXKO chow=5), HFD (**D**, fl/Y HFD=6, UTXKO HFD=5) or challenged with cold (**E**, n=6/group). *indicates statistical significance between the two groups by Mann-Whitney's nonparametric U test.

All data are expressed as mean ± SEM.

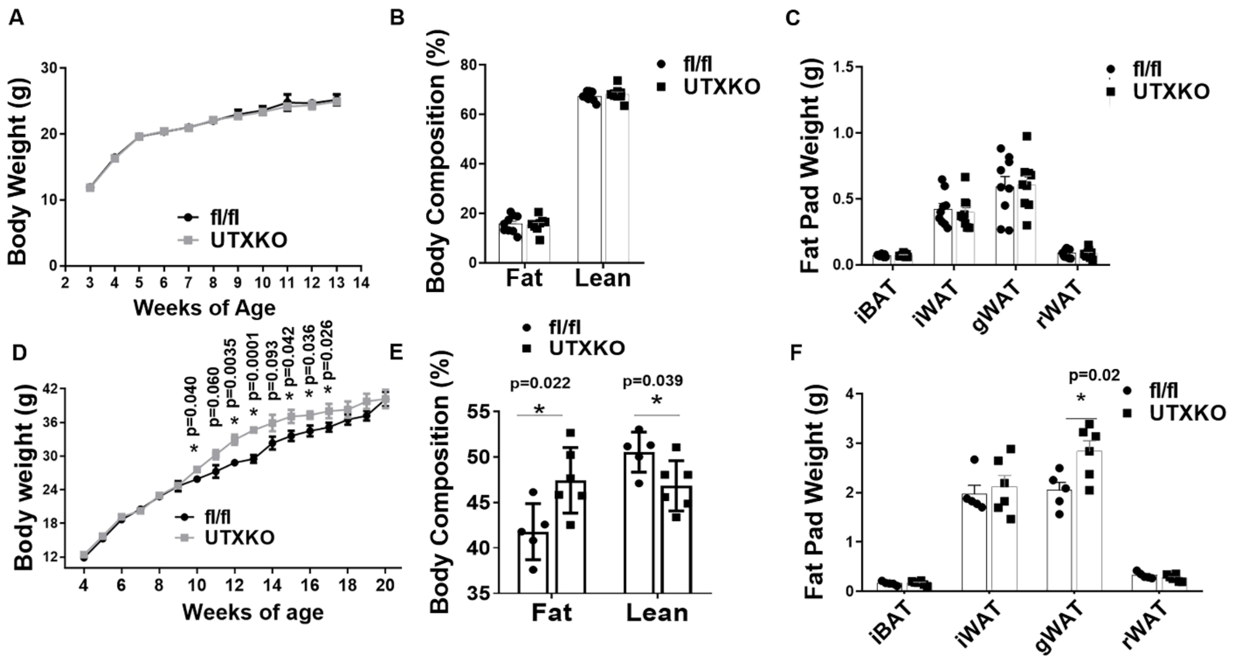

Supplemental figure 6. Metabolic phenotypes in female UTXKO and fl/fl littermate control mice fed chow or HFD.

(**A**)-(**C**) Body weight (**A**), body composition (**B**) and fat pad weight (**C**) in female UTXKO and fl/fl mice fed chow diet (n=9/group).

(**D**)-(**F**) Body weight (**D**, n=6/group), body composition (**E**, n=5 for fl/fl, and 6 for UTXKO) and fat pad weight (**F**, n=5 for fl/fl, and 6 for UTXKO) in female UTXKO and fl/fl mice fed HFD.

All data are expressed as mean ± SEM. *indicates statistical significance between fl/fl and UTXKO by two-tailed unpaired Student's t-test.

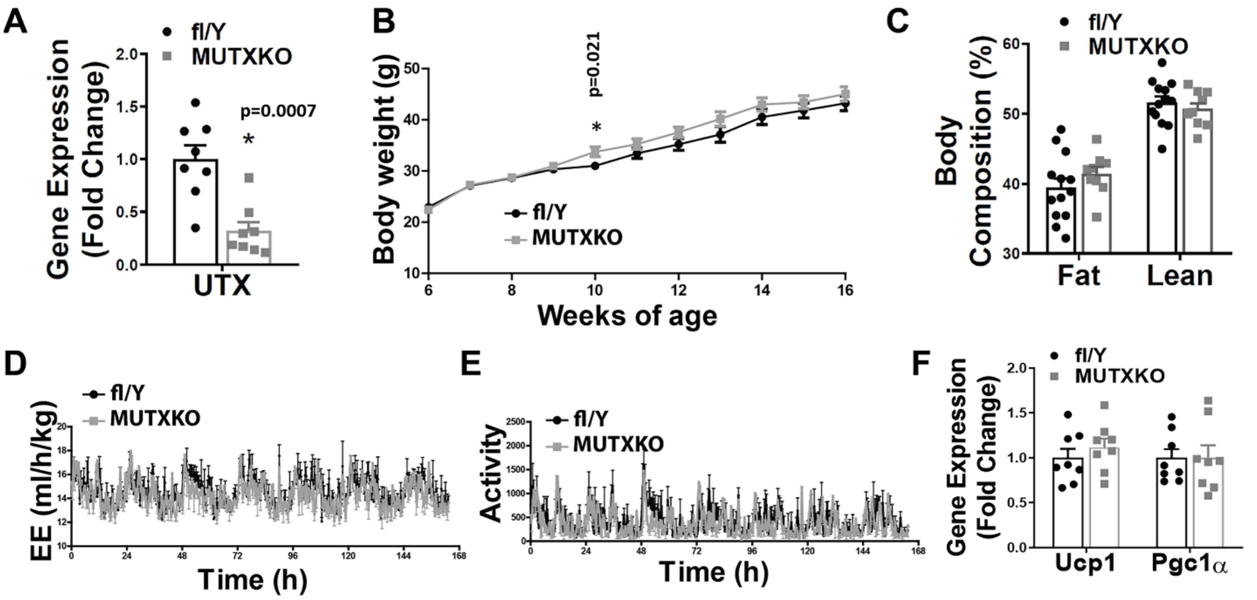

Supplemental figure 7. Mice with *Utx* deficiency in *Myf5*-expressing precursor cells (MUTXKO) have normal energy homeostasis and brown fat thermogenic gene expression when fed HFD diet. Male MUTXKO and fl/Y littermate control mice were put on HFD when they were 6 weeks of age.

(**A**) *Utx* mRNA level in iBAT of male MUTXKO and fl/Y mice (n=8/group).

(**B**)-(**C**) Body weight growth curve (**B**, n=12 for fl/Y, and 9 for MUTXKO), and Body composition (**C**, n=13 for fl/Y, and 9 for MUTXKO) of male MUTXKO mice and fl/Y mice on HFD diet.

(**D**)-(**E**) Energy expenditure (EE)(**D**), and Locomotor activity (**E**) in male MUTXKO and fl/Y mice on HFD (n=4/group).

(**F**) *Ucp1* and *Pgc1α* expression in iBAT of male MUTXKO and fl/Y mice on HFD (n=8/group).

All data are expressed as mean ± SEM. *indicates statistical significance between fl/Y and MUTXKO by two-tailed unpaired Student's t-test.

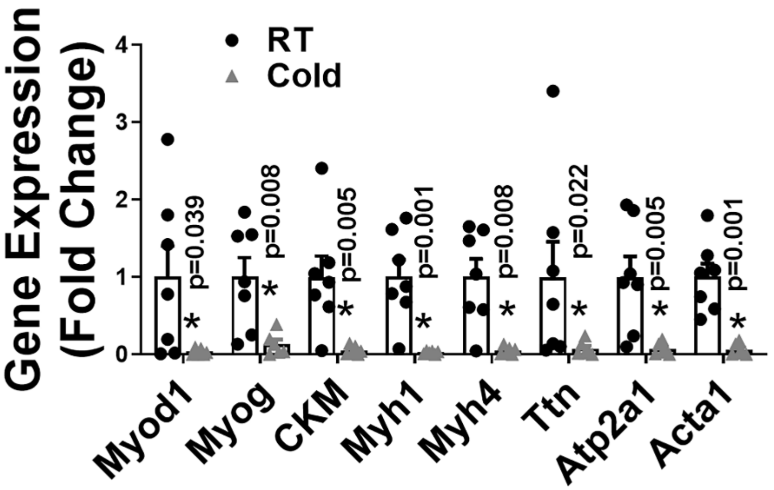

Supplemental Figure 8. Myogenic marker gene expression is down-regulated in iBAT from wild type C57BL/6J mice after a 7-day 5°C cold challenge (n=7 for RT and 6 for Cold). All data are expressed as mean ± SEM. *indicates statistical significance by Mann-Whitney's nonparametric U test.

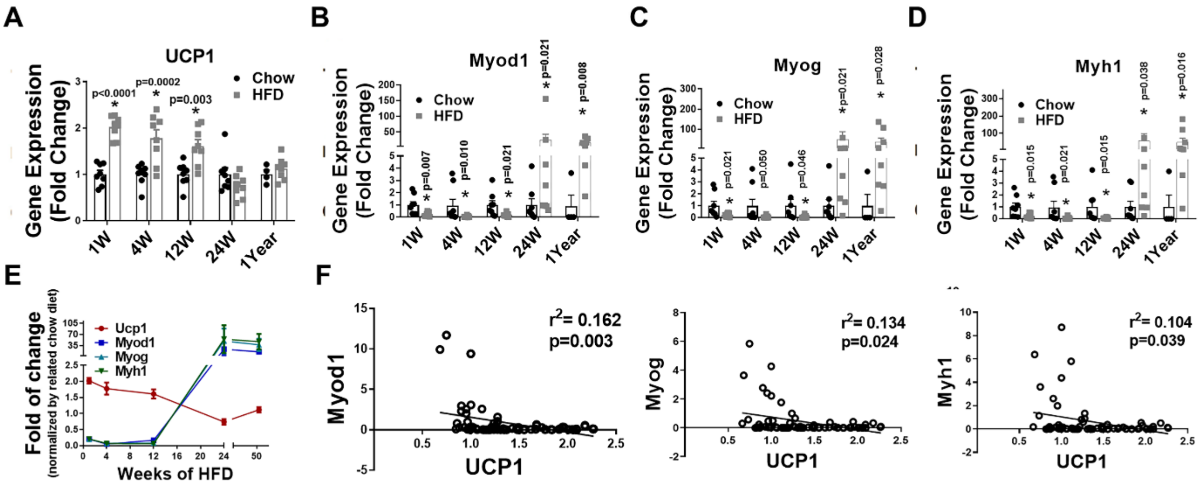

Supplemental figure 9. The expression of *Ucp1* and myogenic marker genes are negatively correlated in iBAT. Male C57BL/6J mice were put on either chow or HFD diet when they were 6 weeks old.

(**A**)-(**D**) Quantitative RT-PCR analysis of *Ucp1* (**A**), *Myod1* (**B**), *Myog* (**C**), and *Myh1* (**D**) expression in iBAT of mice fed chow or HFD for 1 week, 4 weeks, 12 weeks, 24 weeks and 1 year (n=4 for 1 year chow group, and n=8/group for all other groups). *indicates statistical significance analyzed by two-tailed unpaired Student's t-test in (**A**) and Mann-Whitney's nonparametric U test in (**B**), (**C**) and (**D**).

(**E**) Quantitative RT-PCR analysis of *Ucp1* and myogenic marker gene expression patterns in iBAT of HFD-fed mice for 1 week, 4 weeks, 12 weeks, 24 weeks and 1 year (n=8/group).

(**F**) Negative correlations between *Ucp1* and myogenic marker gene expression in iBAT of mice fed chow or HFD for 1 week, 4 weeks, 12 weeks, 24 weeks and 1 year (n=66 for the correlation between *Ucp1* and *Myod1*, n=71 for the correlation between *Ucp1* and *Myog*, and between *Ucp1* and *Myh1*). Correlation between *Ucp1* and myogenic gene expression was analyzed by Spearman's rank correlation coefficient test, p=0.003 between *Ucp1* and *Myod1* gene expression, p=0.024 between *Ucp1* and *Myog* gene expression, and p=0.039 between *Ucp1* and *Myh1* gene expression.

All data are expressed as mean ± SEM.

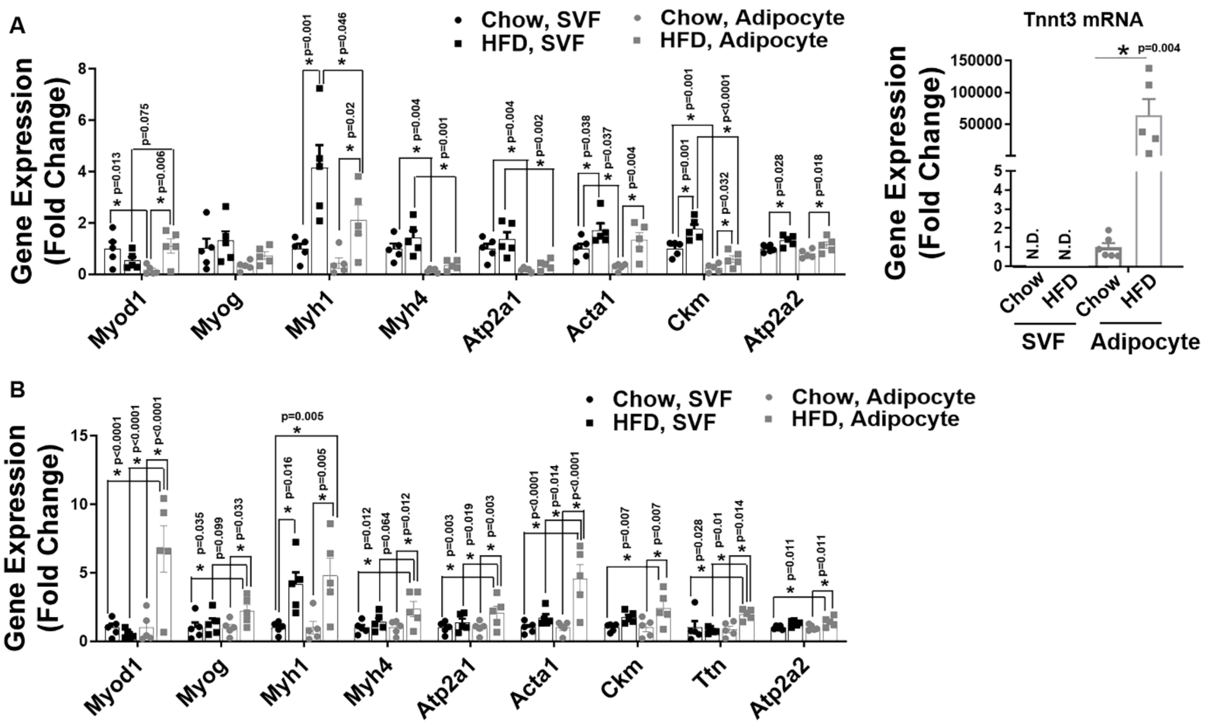

Supplemental Figure 10. Myogenic gene expression in SVF and adipocytes isolated from C57BL/6J mice fed chow or HFD for 24 weeks.

(**A**) Myogenic gene expression in SVF and adipocytes isolated from C57BL/6J mice fed chow or HFD for 24 weeks. n=5/group. For Tnn3, n=6 for Chow adipocytes and n=5 for HFD adipocytes. Gene expression was normalized to the level of Chow SVF. N.D.=not detected. Left panel: *indicates statistical significance analyzed by one-way ANOVA followed by Fisher's LSD multiple comparisons test. *Myod1*, F(3,16)=4.192, p=0.023; *Myog*, F(3,16)=2.132, p=0.14; *Myh1*, F(3,16)=8.806, p=0.001; *Myh4*, F(3,16)=10.50, p<0.0001; *Atp2a1*, F(3,16)=9.490, p<0.0001; *Acta1*, F(3,16)=7.385, p=0.003; *Ckm*, F(3,16)=24.446, p<0.0001; *Atp2a2*, F(3,16)=5.682, p=0.008. Right panel: *indicates statistical significance analyzed by Mann-Whitney's nonparametric U test.

(**B**) Myogenic gene expression in SVF and adipocytes isolated from C57BL/6J mice fed chow or HFD for 24 weeks. n=5/group. Gene expression was normalized to the level of either Chow SVF or chow HFD in each of the SVF or adipocyte group. *indicates statistical significance analyzed by one-way ANOVA followed by Fisher's LSD multiple comparisons test. *Myod1*, F(3,16)=10.943, p<0.0001; *Myog*, F(3,16)=2.413, p=0.11; *Myh1*, F(3,16)=6.025, p=0.006; *Myh4*, F(3,16)=3.612, p=0.036; *Atp2a1*, F(3,16)=5.682, p=0.008; *Acta1*, F(3,16)=8.971, p=0.001; *Ckm*, F(3,16)=4.447, p=0.019; *Ttn*, F(3,16)=3.699, p=0.034; *Atp2a2*, F(3,16)=4.258, p=0.022.

All data are expressed as mean ± SEM.

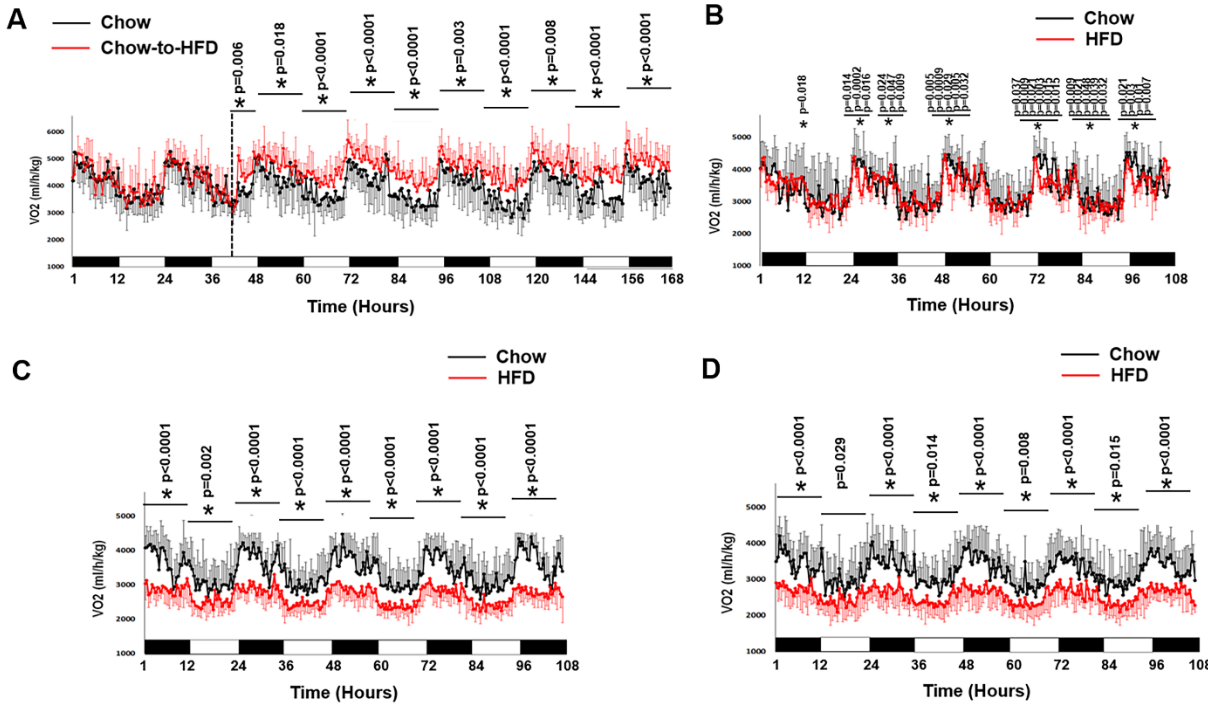

Supplemental figure 11. Oxygen consumption in wild type C57BL/6J mice fed a regular chow or HFD. Male C57BL/6J mice were put on either chow or HFD diet when they were 6 weeks old.

(**A**) Oxygen consumption in 6-week-old wild type C57BL/6J mice on chow diet or in 6-week-old C57BL/6J mice switching from chow to HFD. The dotted line indicates the time point when diet was switched from chow to HFD (n=8/group). *indicates statistical significance between the two groups by ANOVA with repeated measures. Time (41.85-48h), $F(1,12)=11.085$, $p=0.006$; Time (48-60h), $F(1,12)=7.541$, $p=0.018$; Time (60-72h), $F(1,12)=58.218$, $p<0.0001$; Time (72-84h), $F(1,12)=26.367$, $p<0.0001$; Time (84-96h), $F(1,12)=44.103$, $p<0.0001$; Time (96-108h), $F(1,12)=13.285$, $p=0.003$; Time (108-120h), $F(1,12)=35.549$, $p<0.0001$; Time (120-132h), $F(1,12)=9.946$, $p=0.008$; Time (132-144h), $F(1,12)=100.366$, $p<0.0001$; Time (144-156h), $F(1,12)=31.757$, $p<0.0001$.

(**B**)-(**D**) Oxygen consumption in wild type C57BL/6J mice fed a regular chow or HFD for 4 weeks (**B**), 12 weeks (**C**) and 24 weeks (**D**) (n=8/group).

In (**C**) and (**D**), *indicates statistical significance between the two groups by ANOVA with repeated measures. In (**C**), Time (0-12h), $F(1,12)=77.416$, $p<0.0001$; Time (12-24h), $F(1,12)=14.885$, $p=0.002$; Time (24-36h), $F(1,12)=115.218$, $p<0.0001$; Time (36-48h), $F(1,12)=23.504$, $p<0.0001$; Time (48-60h), $F(1,12)=68.6$, $p<0.0001$; Time (60-72h), $F(1,12)=40.186$, $p<0.0001$; Time (72-84h), $F(1,12)=79.507$, $p<0.0001$; Time (84-96h), $F(1,12)=21.537$, $p<0.0001$; Time (96-108h), $F(1,12)=79.3$, $p<0.0001$.

In (**D**), Time (0-12h), $F(1,12)=48.97$, $p<0.0001$; Time (12-24h), $F(1,12)=6.108$, $p=0.029$; Time (24-36h), $F(1,12)=30.184$, $p<0.0001$; Time (36-48h), $F(1,12)=8.239$, $p=0.014$; Time (48-60h), $F(1,12)=27.085$, $p<0.0001$; Time (60-72h), $F(1,12)=10.102$, $p=0.008$; Time (72-84h), $F(1,12)=33.658$, $p<0.0001$; Time (84-96h), $F(1,12)=7.994$, $p=0.015$; Time (96-108h), $F(1,12)=23.014$, $p<0.0001$.

For (**B**), there was no statistical significance between the two groups when analyzed by ANOVA with repeated measures at every 12 hours interval. *indicates statistical significance at individual time points analyzed by two-tailed unpaired Student's t-test.
All data are expressed as mean ± SEM.

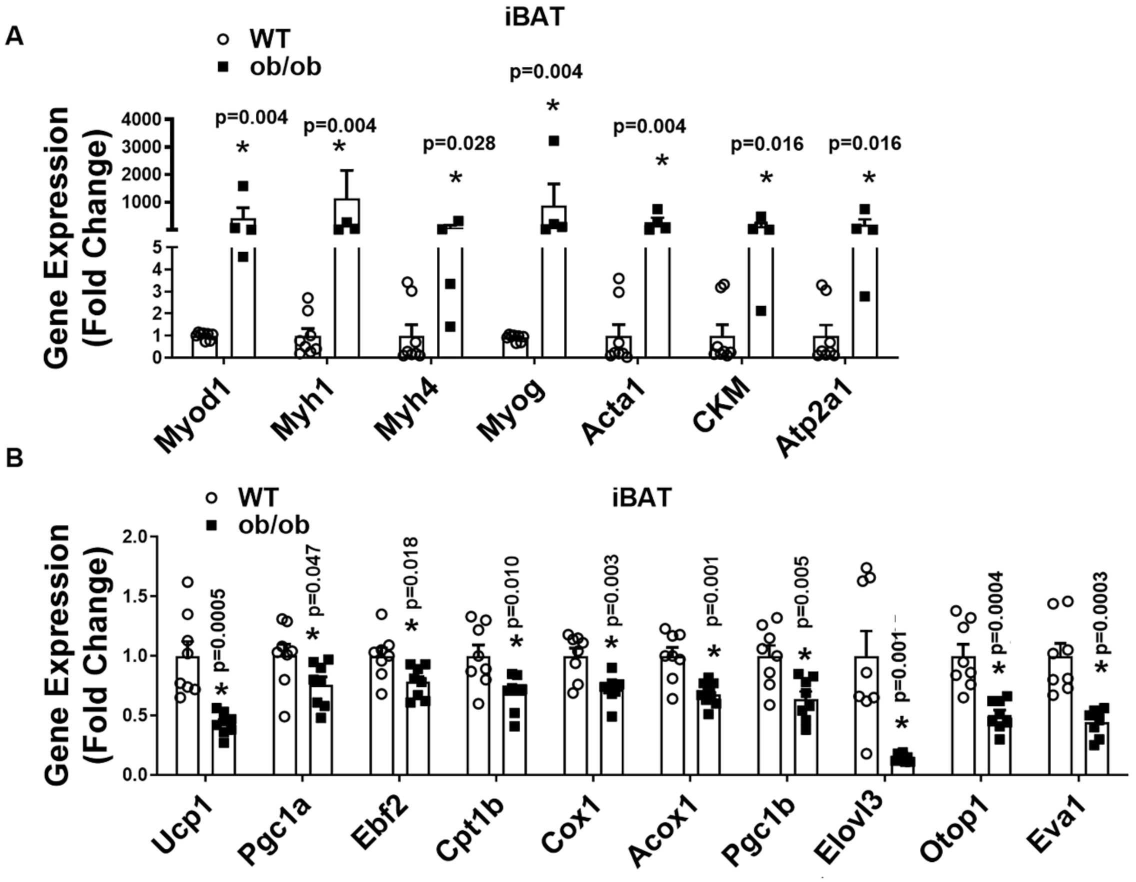

Supplemental figure 12. Myogenic (**A**) or BAT (**B**) specific gene expression in 3-month-old *ob/ob* and their WT Littermate control mice. In (**A**), n=8 for WT and n=4 for *ob/ob*. *indicates statistical significance between the two groups analyzed by Mann-Whitney's nonparametric U test. in (**B**), n=8/group, *indicates statistical significance between the two groups analyzed by two-tailed unpaired Student's t-test. All data are expressed as mean ± SEM.

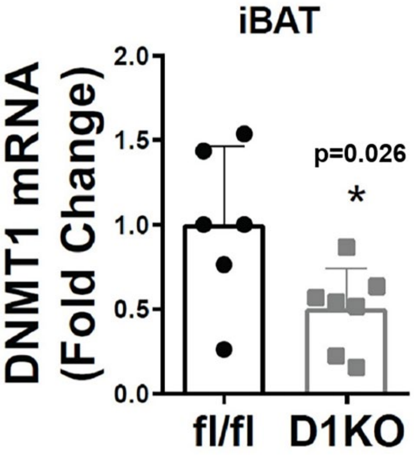

Supplemental Figure 13. Dnmt1 expression in iBAT of D1KO and fl/fl littermate control mice (n=6 for fl/fl and 7 for D1KO).

All data are expressed as mean ± SEM. *indicates statistical significance by two-tailed unpaired Student's t-test.

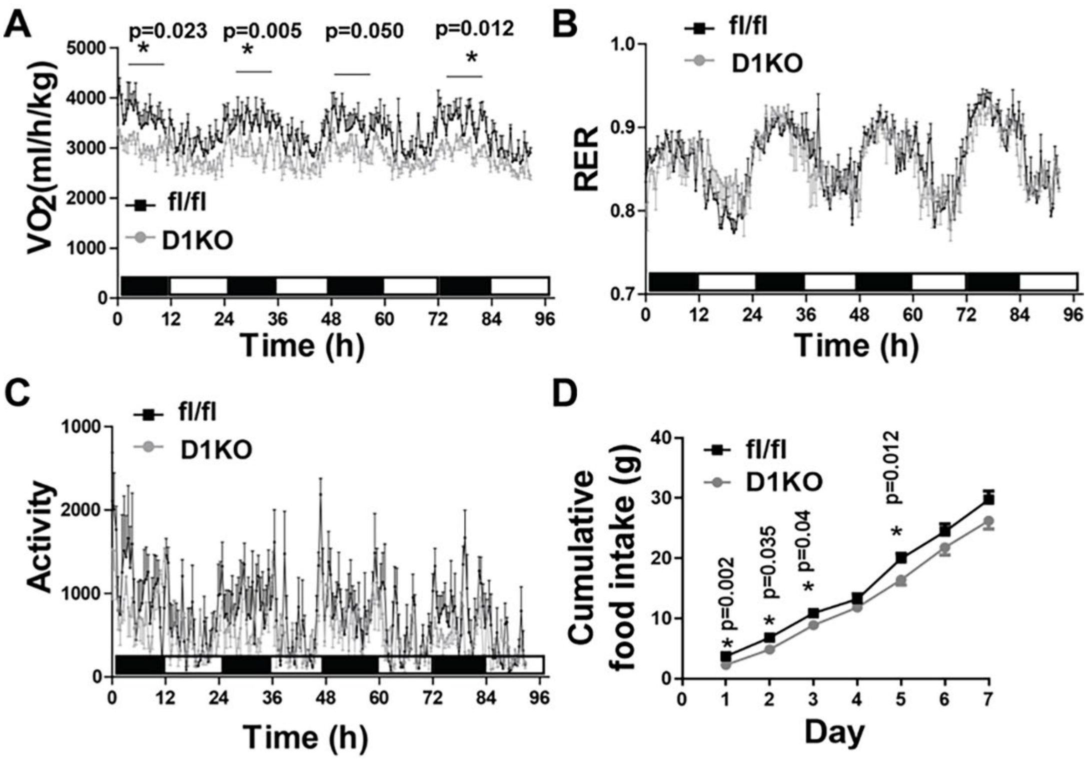

Supplemental figure 14. Metabolic characterization of female D1KO and fl/fl mice on chow diet. (**A**)-(**D**) Oxygen consumption (**A**), RER (**B**), Locomotor activity (**C**), and Cumulative food intake (**D**) in female D1KO mice on chow diet. For **A**-**C**, n=4/group; for **D**, n=6 for fl/fl and 8 for D1KO. In (**A**), *indicates statistical significance between the two groups analyzed by ANOVA with repeated measures, time 0-12 hours, $F(1,6)=9.163$, $p=0.023$; time 24-36 hours, $F(1,6)=19.341$, $p=0.005$; time 48-60 hours, $F(1,6)=5.976$, $p=0.050$; time 72-84 hours, $F(1,6)=12.619$, $p=0.012$. In (**D**), *indicates statistical significance between the two groups analyzed by two-tailed unpaired Student’s t-test.
All data are expressed as mean ± SEM.

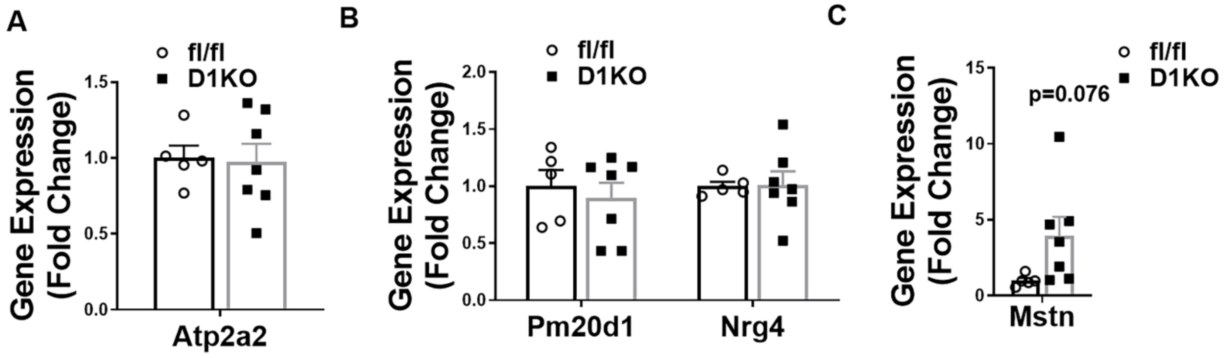

Supplemental figure 15. Gene expression analysis of *Atp2a2* (**A**), *Pm20d1* and *Nrg4* (**B**), and *Mstn* (**C**) in iBAT of chow-fed female D1KO and fl/fl littermate control mice. n=5 for fl/fl, 7 for D1KO. All data are expressed as mean ± SEM. Statistical analysis was performed by two-tailed unpaired Student's t-test.

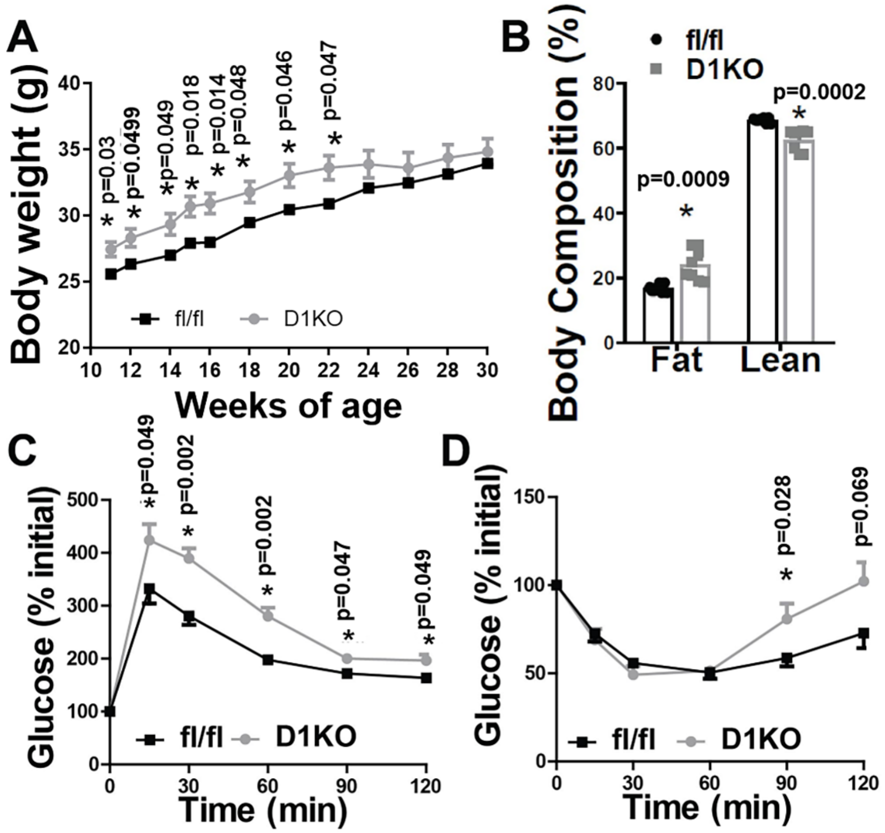

Supplemental figure 16. Metabolic characterization of male D1KO and their control fl/fl mice on chow diet.

(**A**)-(**D**) Body weight growth curve (**A**, n=5 for fl/fl and 8 for D1KO), Body composition (**B**, n=8/group), GTT (**C**, n=5 for fl/fl and 8 for D1KO), and ITT (**D**, n=5 for fl/fl and 8 for D1KO) in male D1KO and their control fl/fl mice on chow diet.

All data are expressed as mean ± SEM. *indicates statistical significance between fl/fl and D1KO by two-tailed unpaired Student's t-test.

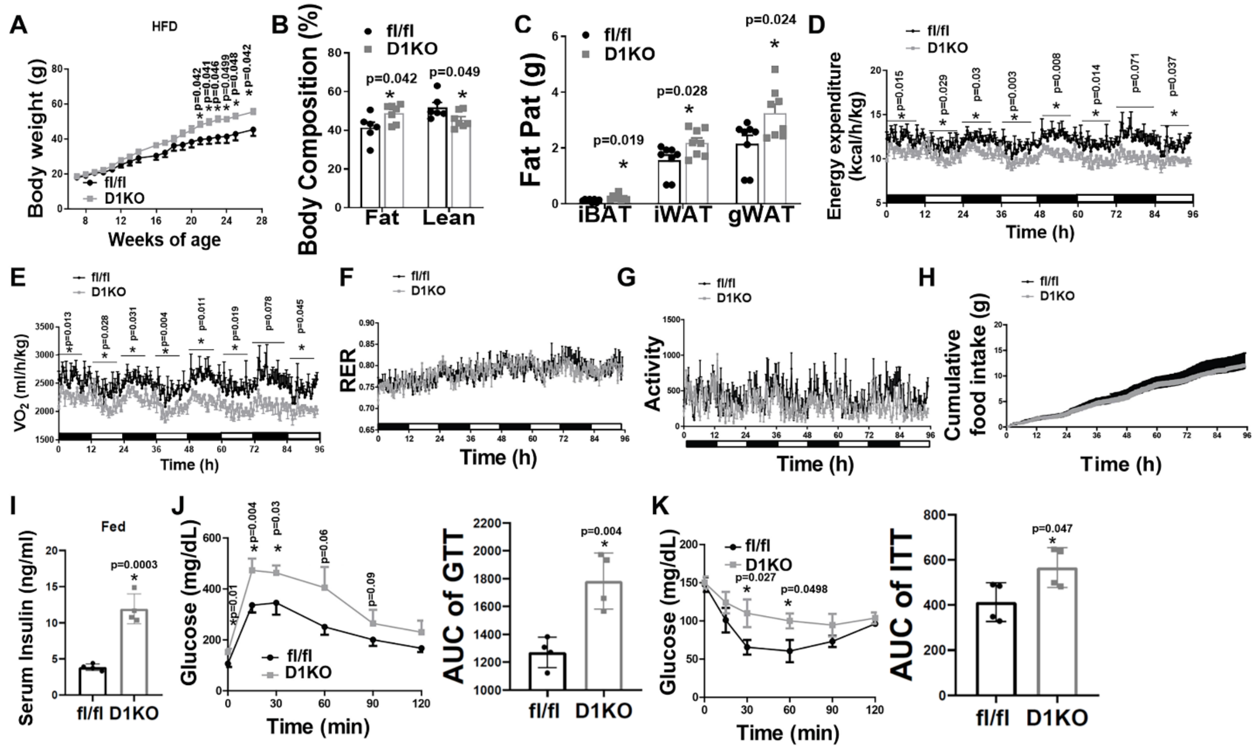

Supplemental figure 17. Female D1KO mice are susceptible to DIO. Female D1KO and fl/fl were put on HFD diet when they were 6 weeks old.

(**A**)-(**C**) Body weight growth (**A**, n=4/group), Body composition (**B**, n=6/group), and Fat pad weight (**C**, n=8/group) of female D1KO mice and their littermate fl/fl controls fed HFD. *indicates statistical significance between the two groups by unpaired two-tailed Student's t-test.

(**D**)-(**H**) Energy expenditure (**D**), Oxygen consumption (**E**), RER (**F**), Locomotor activity (**G**), and Cumulative food intake (**H**) of female D1KO mice and their littermate fl/fl controls on HFD (n=4/group). For left panel in (**D**) and (**E**), *indicates statistical significance between the two groups by One-Way ANOVA with repeated measures. In (**D**), time 0-12 hours, $F(1,6)=11.528$, $p=0.015$; time 12-24 hours, $F(1,6)=8.212$, $p=0.029$; time 24-36 hours, $F(1,6)=8.023$, $p=0.03$; time 36-48 hours, $F(1,6)=24.505$, $p=0.003$; time 48-60 hours, $F(1,6)=15.466$, $p=0.008$; time 60-72 hours, $F(1,6)=11.626$, $p=0.014$; time 72-84 hours, $F(1,6)=4.803$, $p=0.071$, time 84-96 hours, $F(1,6)=7.092$, $p=0.037$. In (**E**), time 0-12 hours, $F(1,6)=11.971$, $p=0.013$; time 12-24 hours, $F(1,6)=8.235$, $p=0.028$; time 24-36 hours, $F(1,6)=7.909$, $p=0.031$; time 36-48 hours, $F(1,6)=20.564$, $p=0.004$; time 48-60 hours, $F(1,6)=13.091$, $p=0.011$; time 60-72 hours, $F(1,6)=10.221$, $p=0.019$; time 72-84 hours, $F(1,6)=4.509$, $p=0.078$; time 84-96 hours, $F(1,6)=6.344$, $p=0.045$. For right panel in (**D**) and (**E**), *indicates statistical significance between the two groups by unpaired two-tailed Student's t-test.

(**I**)-(**K**) Fed insulin levels (**I**), GTT (**J**), and ITT (**K**) of female D1KO mice and their littermate fl/fl controls on HFD (n=4/group). *indicates statistical significance between the two groups by unpaired two-tailed Student's t-test.

All data are expressed as mean ± SEM.

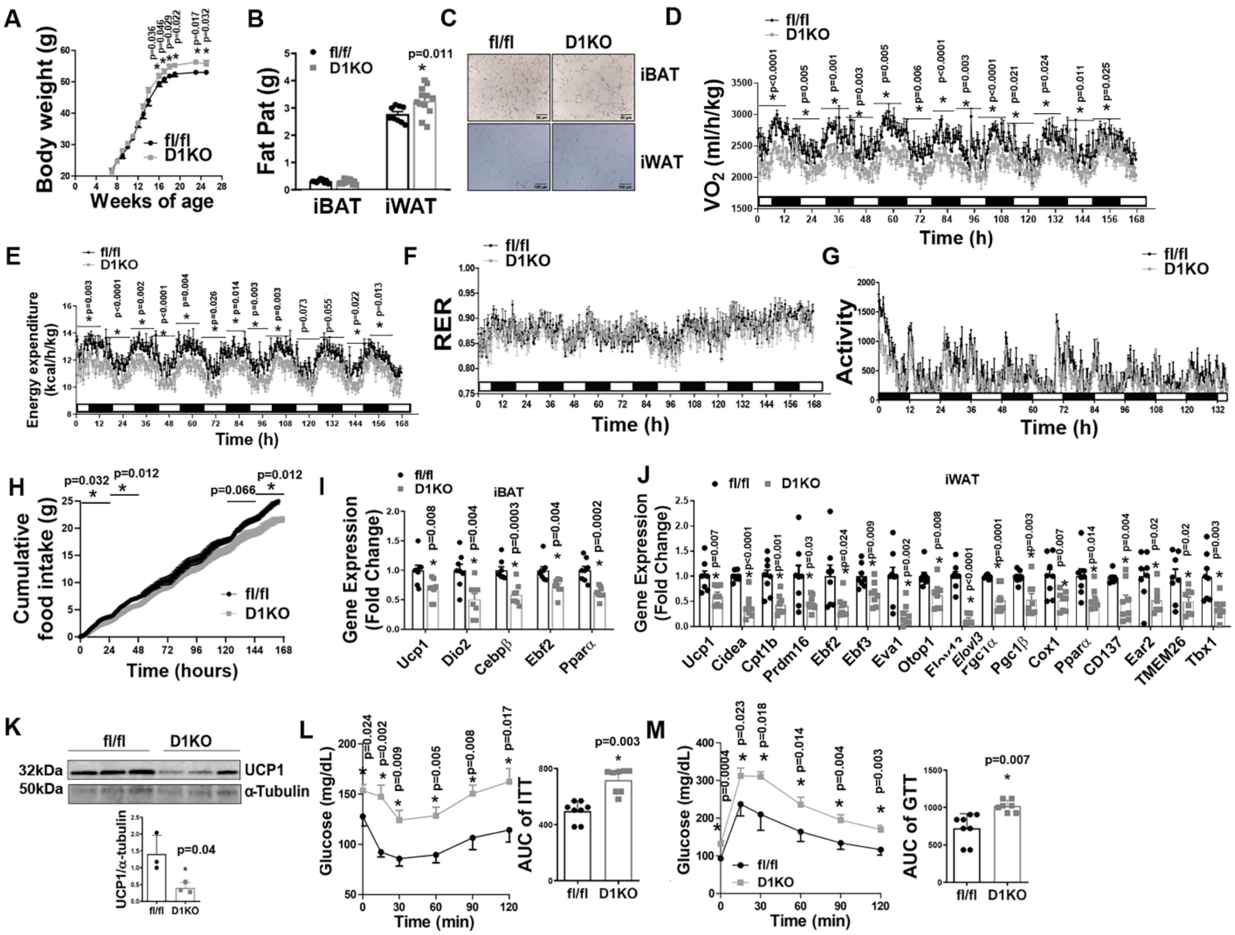

Supplemental figure 18. Metabolic characterization of male D1KO and their control fl/fl mice on HFD. Male D1KO and fl/fl mice were put on HFD diet when they were 6 weeks of age.

(**A**)-(**B**) Body weight growth curve (**A**, n=6 for fl/fl and 5 for D1KO) and Fat pad weight (**B**, n=9 for fl/fl and 12 for D1KO) in male D1KO and fl/fl mice on HFD. *indicates statistical significance between the two groups by unpaired two-tailed Student's t-test.

(**C**) Representative H&E staining of iBAT and iWAT in male D1KO and fl/fl mice on HFD (n=3 replicates per group).

(**D**)-(**H**) Oxygen consumption (**D**, n=4/group), Energy expenditure (**E**, n=8/group), RER (**F**, n=4/group), Locomotor activity (**G**, n=4/group), and Cumulative food intake (**H**, n=4/group) in male D1KO and fl/fl mice on HFD. *indicates statistical significance between the two groups by One-Way ANOVA with repeated measures.

In (**D**), time 0-12 hours, $F(1,6)=37.841$, $p<0.0001$; time 12-24 hours, $F(1,6)=18.319$, $p=0.005$; time 24-36 hours, $F(1,6)=30.925$, $p=0.001$; time 36-48 hours, $F(1,6)=24.505$, $p=0.003$; time 48-60 hours, $F(1,6)=18.941$, $p=0.005$; time 60-72 hours, $F(1,6)=16.78$, $p=0.006$; time 72-84 hours, $F(1,6)=41.852$, $p<0.0001$; time 84-96 hours, $F(1,6)=22.729$, $p=0.003$; time 96-108 hours, $F(1,6)=44.918$, $p<0.0001$; time 108-120 hours, $F(1,6)=9.602$, $p=0.021$; time 120-132 hours, $F(1,6)=9.033$, $p=0.024$; time 132-144 hours, $F(1,6)=13.070$, $p=0.011$; time 144-156 hours, $F(1,6)=8.865$, $p=0.025$.

In (**E**), time 0-12 hours, $F(1,14)=12.51$, $p=0.003$; time 12-24 hours, $F(1,14)=19.578$, $p<0.0001$; time 24-36 hours, $F(1,14)=13.916$,$p=0.002$; time 36-48 hours, $F(1,14)=25.796$,$p<0.0001$; time 48-60 hours, $F(1,14)=11.584$,$p=0.004$; time 60-72 hours, $F(1,14)=6.167$, $p=0.026$; time 72-84 hours, $F(1,14)=7.817$, $p=0.014$; time 84-96 hours, $F(1,14)=13.148$, $p=0.003$; . time 96-108 hours, $F(1,14)=12.291$, $p=0.003$; time 108-120 hours, $F(1,14)=3.771$, $p=0.073$; time 120-132 hours, $F(1,14)=4.383$, $p=0.055$; time 132-144 hours, $F(1,14)=6.143$, $p=0.022$; time 144-156 hours, $F(1,14)=7.986$, $p=0.013$.

In (**H**), Time (0-24h), $F(1,6)=7.718$, $p=0.032$; Time (24-48h), $F(1,6)=12.882$, $p=0.012$; Time (48-72h), $F(1,6)=3.777$, $p=0.1$; Time (72-96h), $F(1,6)=2.277$, $p=0.182$; Time (96-120h), $F(1,6)=3.193$, $p=0.124$; Time (120-144h), $F(1,6)=5.011$, $p= 0.066$; Time (144-168h), $F(1,6)=12.757$, $p=0.012$.

(I)-(**J**) Quantitative PCR analysis of thermogenic gene expression in iBAT (**I**) and iWAT (**J**) of male D1KO and fl/fl mice on HFD (n=8/group). *indicates statistical significance between the two groups by unpaired two-tailed Student's t-test.

(**K**) Immunoblotting of UCP1 protein in iBAT of male D1KO and fl/fl mice on HFD (n=3/group). *indicates statistical significance between the two groups by unpaired two-tailed Student's t-test.

(**L**)-(**M**) GTT (**L**) and ITT (**M**) in male D1KO and fl/fl mice on HFD (n=5/group). *indicates statistical significance between the two groups by unpaired two-tailed Student's t-test.

All data are expressed as mean ± SEM.

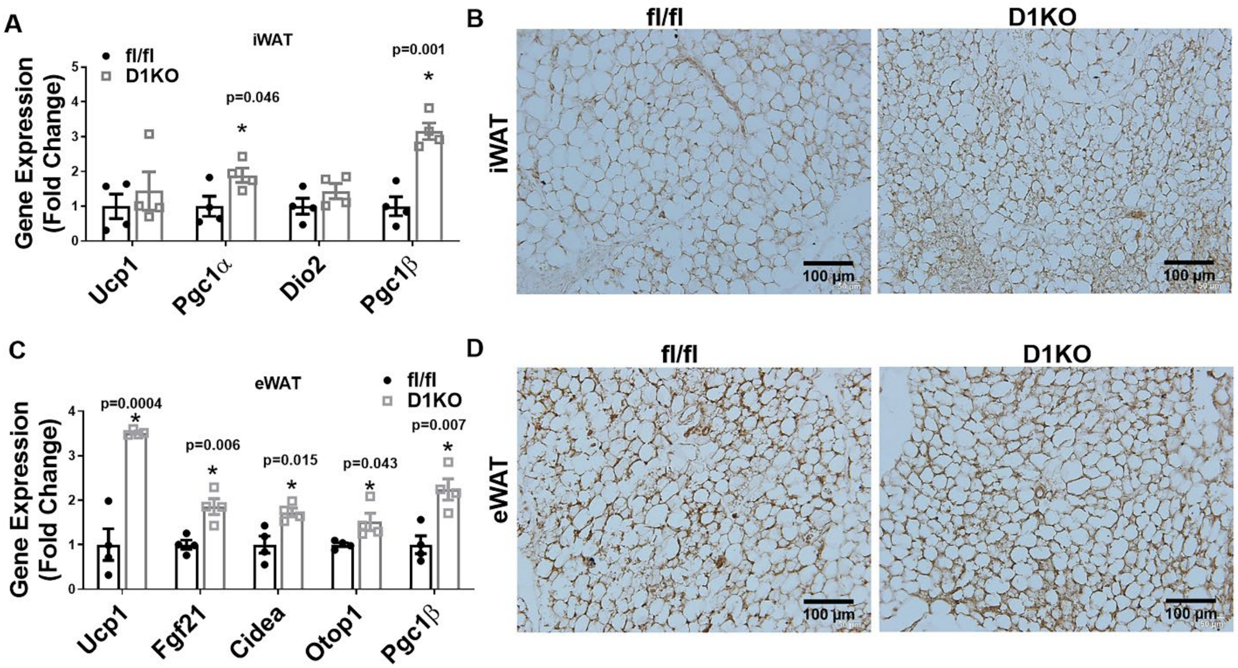

Supplemental figure 19. Characterization of D1KO and fl/fl during cold exposure. Two months old male D1KO and fl/fl mice were subjected to a chronic 7-day cold exposure challenge at 5°C.

(**A**)-(**B**) Quantitative RT-PCR analysis of thermogenic gene expression (n=4/group) (**A**) and representative H&E staining (**B**, 3 replicates/group) in iWAT of male D1KO and fl/fl mice after chronic 7-day cold exposure.

(**C**)-(**D**) Quantitative RT-PCR analysis of thermogenic gene expression (n=4/group) (**C**) and representative H&E staining (**D**, 3 replicates/group) in eWAT of male D1KO and fl/fl mice after chronic 7-day cold exposure.

All data are expressed as mean ± SEM. *indicates statistical significance between the two groups by unpaired two-tailed Student's t-test.

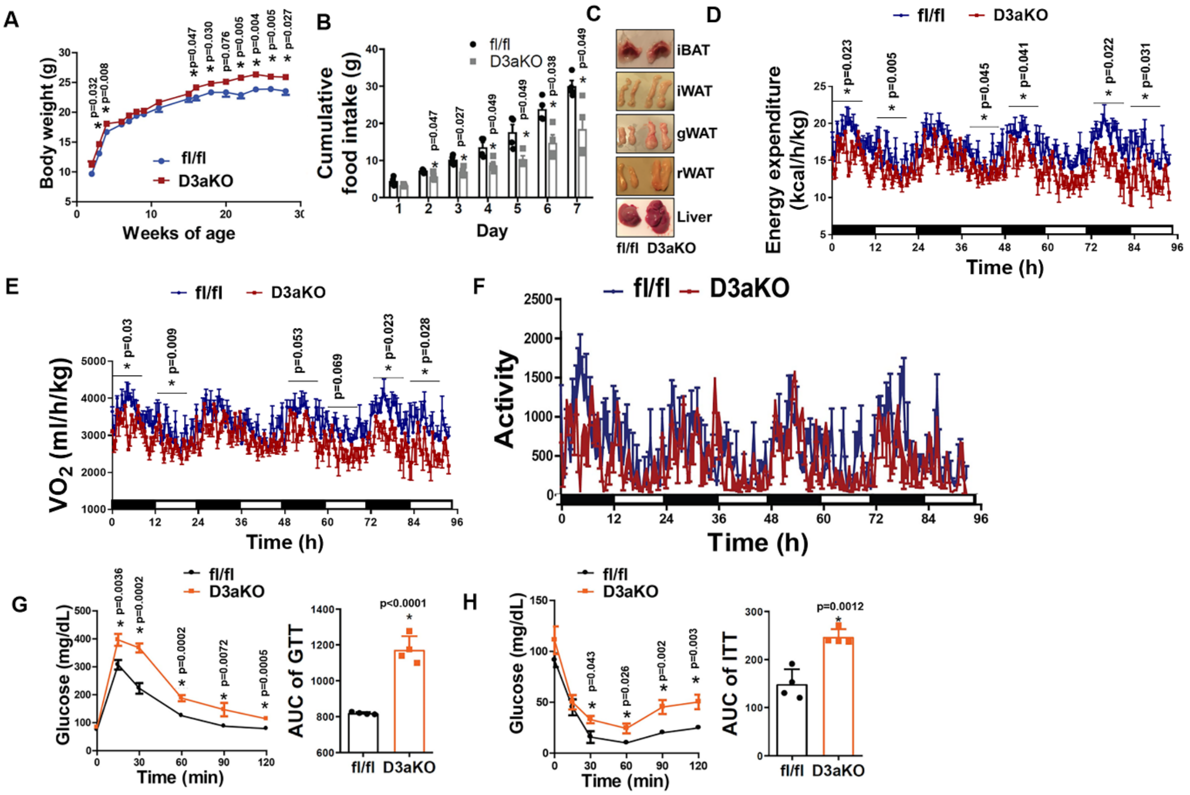

Supplemental figure 20 (Related to Figure 6). Metabolic characterization of D3aKO and their control fl/fl mice on chow diet.

(**A**)-(**B**) Body weight growth curve (**A**, n=4/group) and Food intake (**B**, n=4 for fl/fl and 3 for D3aKO) in D3aKO and their control fl/fl mice on chow diet. *indicates statistical significance between the two groups by unpaired two-tailed Student's t-test.

(**C**) Representative fat depot and liver images in D3aKO and their control fl/fl mice on chow diet (n=4 replicates/group).

(**D**)-(**H**) Energy expenditure (**D**), Oxygen consumption (**E**), Locomotor activity (**F**), GTT (**G**), and ITT (**H**) in D3aKO and their control fl/fl mice on chow diet (n=4/group).

In (**D**), (**E**) and (**F**), *indicates statistical significance between the two groups by one-way ANOVA with repeated measures. In (**D**), time 0-12 hours, F(1,6)=9.188, p=0.023; time 12-24 hours, F(1,6)=19.367, p=0.005; time 24-36 hours, F(1,6)=2.211, p=0.188; time 36-48 hours, F(1,6)=6.333, p=0.045; time 48-60 hours, F(1,6)=6.769, p=0.041; time 60-72 hours, F(1,6)=4.799, p=0.071; time 72-84 hours, F(1,6)=9.362, p=0.021; time 84-96 hours, F(1,6)=7.857, p=0.037.

In (**E**), time 0-12 hours, F(1,6)=7.96, p=0.03; time 12-24 hours, F(1,6)=14.272, p=0.009; time 24-36 hours, F(1,6)=1.758, p=0.233; time 36-48 hours, F(1,6)=5.197, p=0.063; time 48-60 hours, F(1,6)=5.789, p=0.053; time 60-72 hours, F(1,6)=4.883, p=0.069; time 72-84 hours, F(1,6)=9.109, p=0.023; time 84-96 hours, F(1,6)=8.357, p=0.028. In (**G**) and (**H**), *indicates statistical significance between the two groups by unpaired two-tailed Student's t-test.

All data are expressed as mean ± SEM.

A

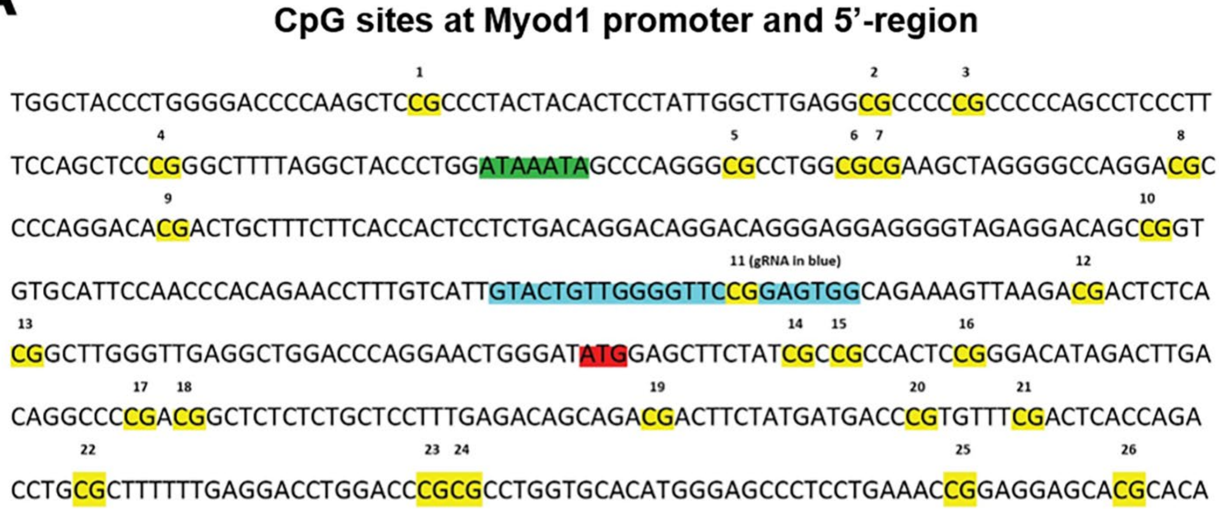

B

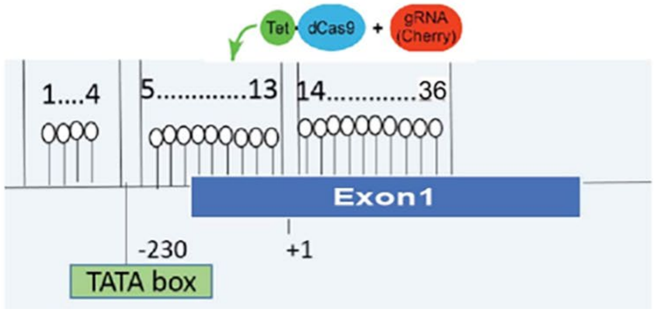

Supplemental figure 21. (**A**) CpG sites at *Myod1* promoter and 5'-region. Yellow highlights indicate CpG sites; green highlight indicates the TATAA-box; red highlight indicates translational start site (ATG); blue-highlighted region indicates the location of guide RNA (gRNA) that was used in the directed methylation targeting approach described in (**B**).

(**B**) Schematic diagram of the strategy to specifically target demethylation at *Myod1* promoter with dCas9-TET1CD and gRNA system.

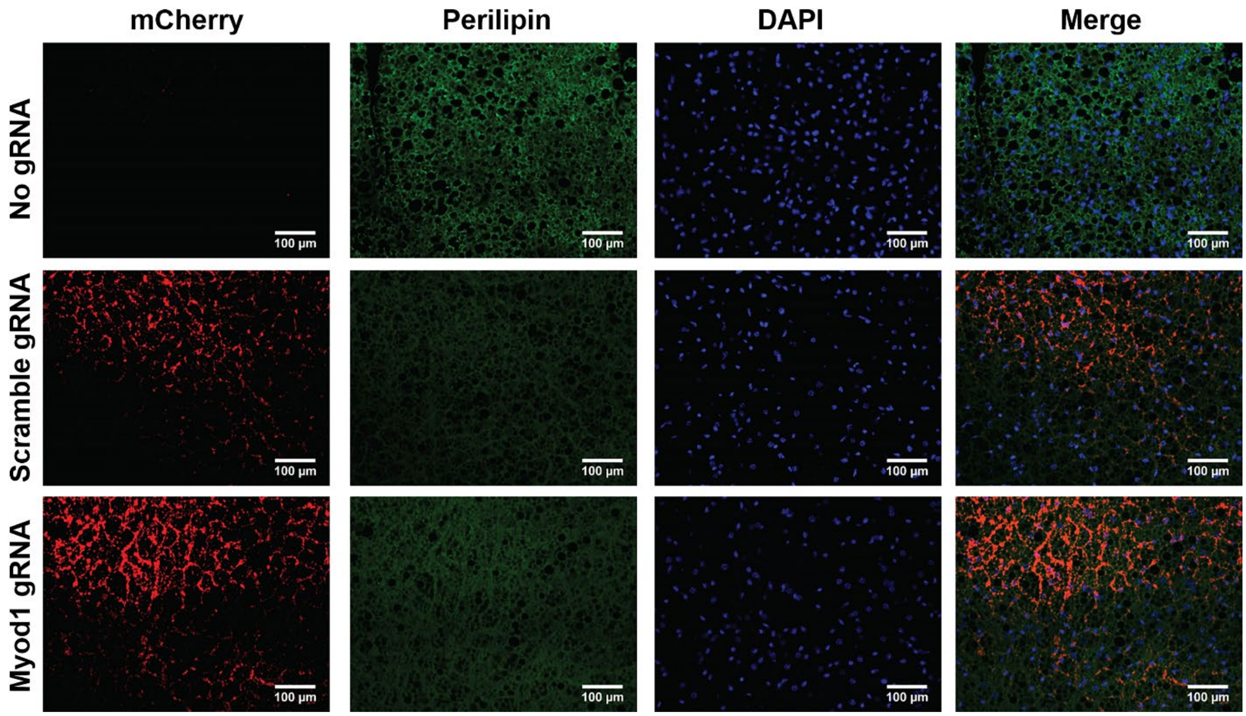

Supplemental figure 22. Representative images indicate IHC staining of mCherry (red), perilipin (green), DAPI (Blue) and merged images from iBAT of lentivirus-injected mice (3 replicates/group). Three-month-old chow-fed male C57BL/6J mice were bilaterally injected with lentiviruses expressing dCas9-TET1CD plus lentiviruses expressing either targeting Myod1-gRNA-mCherry or non-targeting scramble-gRNA-mCherry into iBAT. Tissues were collected 2 months after the injection.

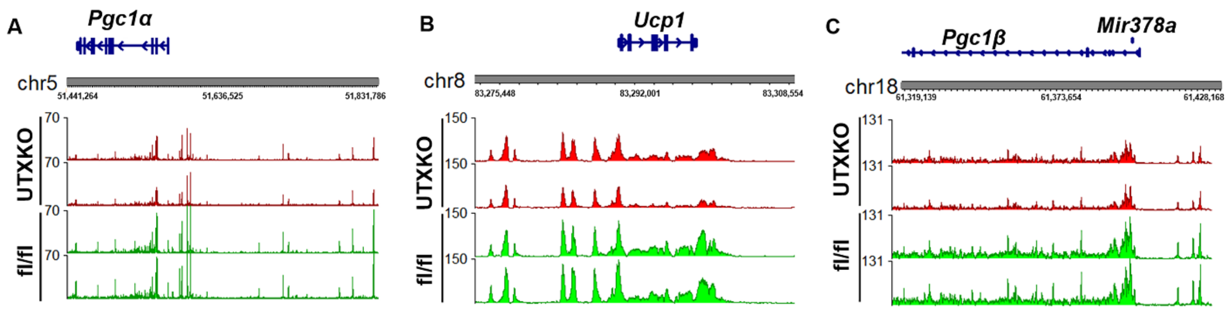

Supplemental Figure 23. ATAC-seq analysis of chromatin accessibility at *Pgc1α* (**A**), *Ucp1* (**B**) and *Pgc1β* (**C**) loci.

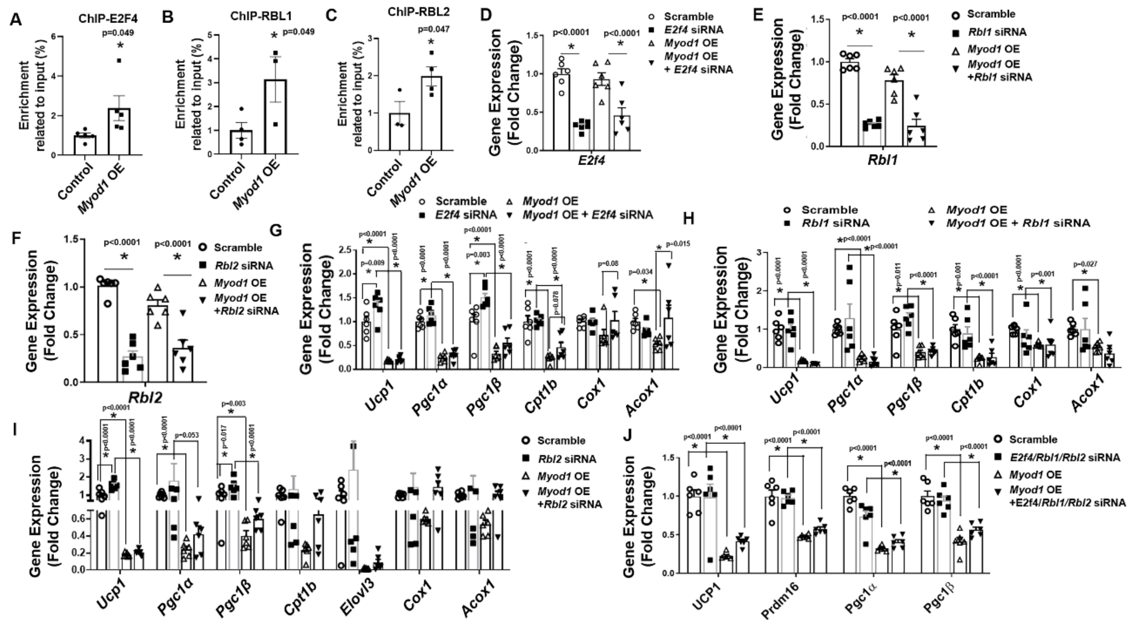

Supplemental Figure 24. Interaction between E2F4/RBL1/RBL2 transcription repressor complex at *Prdm16* promoter.

(**A**)-(**C**) Binding of E2F4 (**A**, n=5/group), RBL1 (**B**, n=4 for Control, and 3 for *Myod1* overexpression (OE)) and RBL2 (**C**, n=3 for Control and 4 for *Myod1* OE) to *Prdm16* promoter in *Myod1*-overexpressed BAT1 brown adipocytes by ChIP assay. *indicates statistical significance by two-tailed unpaired Student's t-test.

(**D**)-(**F**) Knocking down efficiency in *Myod1*-overexpressed BAT1 brown adipocytes with simultaneous knockdown of *E2f4* (**D**), *Rbl1* (**E**) and *Rbl2* (**F**) (n=6/group). *indicates statistical significance between groups with one-way ANOVA followed by Fisher's LSD multiple comparisons tests: F(3,20)=21.190, p<0.001 in (**D**), F(3,20)=46.335, p<0.001 in (**E**); F(3,20)=33.162, p<0.001 in (**F**).

(**G**)-(**J**) BAT-specific gene expression in *Myod1*-overexpressed BAT1 brown adipocytes with simultaneous knockdown of *E2f4* (**G**), *Rbl1* (**H**), *Rbl2* (**I**) and in combination (**J**) (n=6/group). *indicates statistical significance among groups with one-way ANOVA followed by Fisher's LSD multiple comparisons test. In (**G**), F(3,20)=51.141, p<0.0001 for *Ucp1*; F(3,20)=71.342, p<0.0001 for *Pgc1α*; F(3,20)=24.74, p<0.0001 for *Pgc1β*; F(3,20)=21.315, p<0.0001 for *Cpt1b*; F(3,20)=1.522, p=0.24 for *Cox1*; F(3,20)=2.773, p=0.068 for *Acox1*.

In (**H**), F(3,20)=46.335, p<0.001 for *Ucp1*; F(3,20)=31.467, p<0.0001 for *Pgc1α*; F(3,20)=8.475, p=0.001 for *Pgc1β*; F(3,20)=17.768, p<0.0001 for *Cpt1b*; F(3,20)=11.637, p<0.0001 for *Cox1*; F(3,20)=2.635, p=0.078 for *Acox1*.

In (**I**), F(3,20)=82.253, p<0.001 for *Ucp1*; F(3,20)=2.182, p=0.122 for *Pgc1α*; F(3,20)=14.071, p<0.0001 for *Pgc1β*; F(3,20)=1.569, p=0.228 for *Cpt1b*; F(3,20)=1.964, p=0.152 for *Elovl3*; F(3,20)=1.079, p=0.381 for *Cox1*; F(3,20)=1.04, p=0.396 for *Acox1*.

In (**J**), F(3,20)=16.822, p<0.001 for *Ucp1*; F(3,20)=33.361, p<0.0001 for *Prdm16*; F(3,20)=44.25, p<0.0001 for Pgc1α; F(3,20)=26.998, p<0.0001 for *Pgc1β*.

All data are expressed as mean ± SEM.

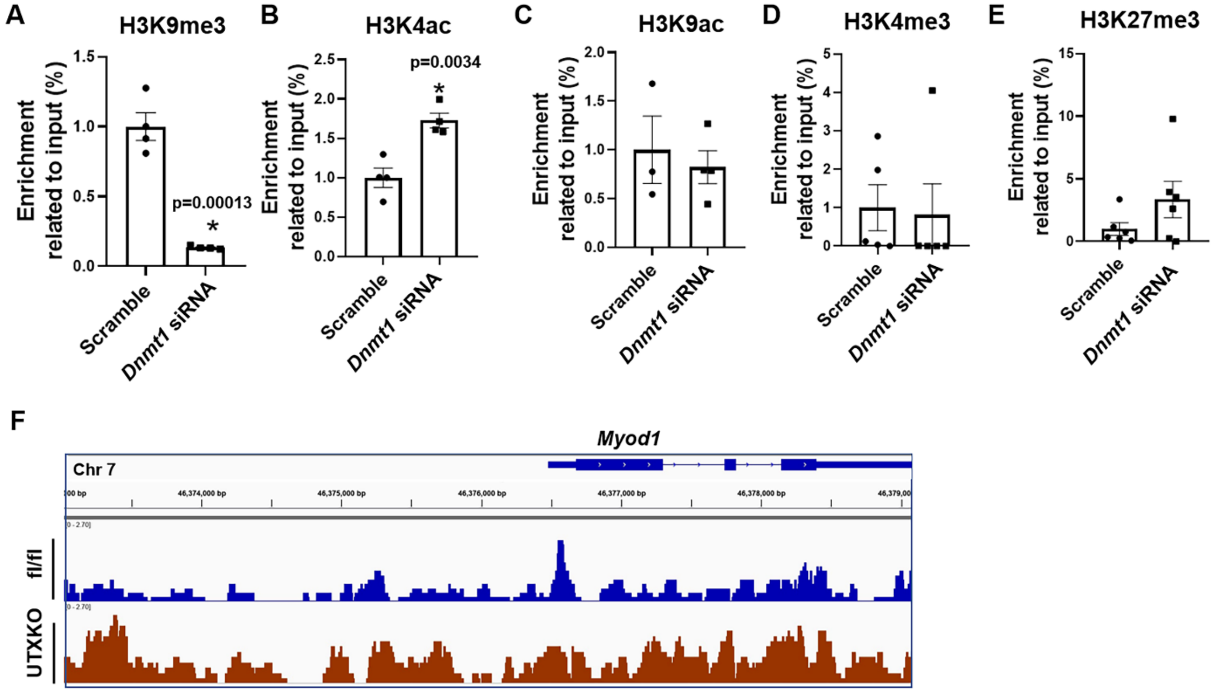

Supplemental Figure 25. The effect of DNMT1-mediated DNA methylation on histone modifications at Myod1 gene locus.

(**A**)-(**E**) H3K9me3 (**A**, n=4/group), H3K4ac (**B**, n=4/group), H3K9ac (**C**, n=3 for Scramble and 4 for *Dnmt1* siRNA), H3K4me3 (**D**, n=5/group) and H3K27me3 (**E**, n=6/group) at *Myod1* promoter in BAT1 brown adipocytes with *Dnmt1* knock down.

(**F**) ATAC-seq analysis of chromatin accessibility at *Myod1* locus.

All data are expressed as mean ± SEM. *indicates statistical significance by two-tailed unpaired Student's t-test.

Supplemental Table 1. Primer/probe sets for gene expression

Gene symbol	Company	Catalog #
Acox1	ABI	Mm01246834_m1
Acta1	ABI	Mm00808218_g1
Atp2a1	ABI	Mm01275320_m1
Cd137	ABI	Mm01268456-m1
Cidea	ABI	Mm00432554_m1
Ckm	ABI	Mm01321487_m1
Cox	ABI	Mm04225243_g1
Cpt1b	ABI	Mm00487191_g1
Dio2	ABI	Mm0051664_m1
Dnmt1	ABI	Mm00599783-g1
Ear2	ABI	Mm04207376_gH
Ebf2	ABI	Mm00438625_m1
Ebf3	ABI	Mm00438642_m1
Elovl3	ABI	Mm01194165_g1
Eva1	ABI	Mm00468397_m1
Fgf21	ABI	Mm00840165_g1
Klf2	ABI	Mm00500486_g1
Klhl13	ABI	Mm00470674_m1
Mfsd2a	ABI	Mm01192208_m1
Myh1	ABI	Mm01332489_m1
Myh4	ABI	Mm01332541_m1
Myod1	ABI	Mm00440387_m1
Myog	ABI	Mm00446194_m1
Nr4a1	ABI	Mm00440945-m1
Nr4a2	ABI	Mm00443060_m1
Nr4a3	ABI	Mm00450071_g1
Otop1	ABI	Mm00554705_m1
Pparγ	ABI	Mm00440945_m1
Prdm16	ABI	Mm00712556_m1
Sik1	ABI	Mm00440317_m1
Ttn	ABI	Mm00621005_m1
Utx	ABI	Mm01283053_m1
E2F4	ABI	Mm00514160_m1
Rbl1	ABI	Mm01250721_m1
Rbl2	ABI	Mm01242468_m1
Nrg4	ABI	Mm00446254_m1
Atp2a2	ABI	Mm01201431_m1
Pm20d1	ABI	Mm01290668_m1
Myostatin	ABI	Mm01254559_m1
miR-133a	ThermoFisher	Assay ID 002246
miR-133b	ThermoFisher	Assay ID 002247
miR-206	ThermoFisher	Assay ID 000510
miR-1	ThermoFisher	Assay ID 002222
U6 snRNA	ThermoFisher	Assay ID 001973

Table 2. Primer/probe sequences for gene expression

Gene	Primer (Forward: 5’-3’)	Primer (Reverse: 5’-3’)	Probe (5’-3’)
**Cyclophilin (*Ppib*)**	GGTGGAGAGCACCAAGACAGA	GCCGGAGTCGACAATGATG	ATCCTTCAGTGGCTTGTCCCGGCT
Cebpβ	CCAAGAAGACGGTGGACAAG	GTGCTGCGTCTCCAGGTT	CCGCATCTTGTACTCGTCGCTCAG
Pgc1α	CATTTGATGCACTGACAGATGGA	CCGTCAGGCATGGAGGAA	CCGTGACCACTGACAACGAGGCC
Pgc1β	AGGAAGCGGCGGGAAA	CTACAATCTCACCGAACACCTCAA	AGAGATTTCGAATGTATACCACACGGCCTTCA
Ucp1	CACCTTCCCGCTGGACACT	CCCTAGGACACCTTTATACCTAATGG	AGCCTGGCCTTCACCTTGGATCTGA

Supplemental Table 3. Antibodies used in Immunoblotting and ChIP-qPCR

Antibody	Company	Catalog #	Application
**DNMT1**	Abcam	Ab87654	WB(1:500)
**DNMT1**	Santa Cruz Biotechnology	Sc20701 or sc-271729	ChIP(2μg/ml)
**GFP**	Aves labs	GFP-1010	IF(1:1000)
**UCP1**	Abcam	ab23841	WB(1:500)
**UCP1**	Abcam	Ab10983	IHC(1:500)
**KDM6A (UTX)**	Abcam	ab36938	ChIP(2μg/ml)
**KDM6A (UTX)**	Bethyl Laboratories	A302-374A	WB(1:500)
**HA**	Cell signaling technology	C29F4	IP(2μg/ml), WB(1:1000)
**H3K27me3**	Abcam	ab6002	ChIP(2μg/ml)
**H3K9me3**	Abcam	ab8898	ChIP(2μg/ml)
**H3K4me3**	Abcam	ab8580	ChIP(2μg/ml)
**acH3K27**	Abcam	ab4729	ChIP(2μg/ml)
**acH3K9**	Abcam	ab4441	ChIP(2μg/ml)
**acH3K4**	Abcam	ab176799	ChIP(2μg/ml)
**mCherry**	Abcam	ab205402	IF(1:1000)
**MyHC**	DSHB	MF20	IF(1:1000)
**Perilipin**	Everest biotech	EB07728	IF(1:1000)
**PRDM16**	Sigma	SAB2900806	WB(1:500), IP(2μg/ml)
**PRDM16**	Sigma	SAB3500989	ChIP(2μg/ml)
**FLAG**	Sigma	F3165	WB(1:1000),IP(2μg/ml)
**E2F-4**	Santa Cruz Biotechnology	sc-398543	WB(1:1000),ChIP(2μg/ml)
**p107**	Santa Cruz Biotechnology	sc-250	WB(1:1000),ChIP(2μg/ml)
**p130**	Santa Cruz Biotechnology	sc-374521	WB(1:1000),ChIP(2μg/ml)
**α-Tubulin**	Santa Cruz Biotechnology	sc-53646	WB(1:500)
**Biotin-SP (long spacer) AffiniPure Donkey Anti-Rabbit IgG (H+L)**	Jackson ImmunoResearch	711-065-152	IHC(2μg/ml)
**Cy™3 AffiniPure Donkey Anti-Rabbit IgG (H+L)**	Jackson ImmunoResearch	711-165-152	IF(2μg/ml)
**Alexa Fluor® 488 ffiniPure Donkey Anti-Chicken IgY (IgG) (H+L)**	Jackson ImmunoResearch	703-545-155	IF(2μg/ml)
**Goat anti-Mouse IgG (H+L) Highly Cross-Adsorbed Secondary Antibody, Alexa Fluor 488**	Invitrogen	A11029	IF(2μg/ml)
**Goat anti-Mouse IgG (H+L) Highly Cross-Adsorbed Secondary**	Invitrogen	A11032	IF(2μg/ml)

**Antibody, Alexa Fluor 594**			
**Goat anti-Mouse IgG (H+L) Highly Cross-Adsorbed Secondary Antibody, Alexa Fluor 680**	Invitrogen	A21058	WB(1:10000)
**Goat anti-Rabbit IgG (H+L) Highly Cross-Adsorbed Secondary Antibody, Alexa Fluor 680**	Invitrogen	A21109	WB(1:10000)

Supplemental Table 4. Primer sequences for pyrosequencing

Primers	Sequence (5'-3')
Myod1-pyroseq-F1	TGGTTATTTTGGGGATTTTAAGTT
Myod1-pyroseq-*R1	TCTACCCCTCCTCCCTAT
Myod1-pyroseq-S1	ATTTTGGGGATTTTAAGTTT
Myod1-pyroseq-F2	GATAGGGAGGAGGGGTAGAGGATA
Myod1-pyroseq-*R2	TCCCAATTCCTAAATCCAACCTCAAC
Myod1-pyroseq-S2	AGGGGTAGAGGATAG
Myod1-pyroseq-F3	GTTTGGGTTGAGGTTGGATTTA
Myod1-pyroseq-*R3	CTCCCATTTCAAAAAAACTCCCATATACAC
Myod1-pyroseq-S3	GGAATTGGGATATGGAG
Myod1-pyroseq-F4	TGGTGTATATGGGAGTTTTTTTGAA
Myod1-pyroseq-*R4	AACCTCATTCACTTTACTCAA
Myod1-pyroseq-S4	ATGGGAGTTTTTTTGAAAT
Myod1-pyroseq-F5	AGTTGGGTTGGATTGTTATGT
Myod1-pyroseq-*R5	ACCACTACCCCTAATCCC
Myod1-pyroseq-S5	ATGTAGGGTTGGAGA
Myod1-pyroseq-F6	GGGTTAGGGATTAGGGGTAG
Myod1-pyroseq-*R6	CCTTACTCCAAAAATCCTCAAACTC
Myod1-pyroseq-S6	GGGATTAGGGGTAGT

F: Forward primer; R: reverse primer; S: sequencing primer.
*Indicates primers with biotin-tag.
