# Supplementary material for: Epigenetic interaction between UTX and DNMT1 regulates diet-induced myogenic remodeling in brown fat
Source: Nat Commun. 2021 Nov 25;12:6838. doi: 10.1038/s41467-021-27141-7 (PMC8617140; doi:10.1038/s41467-021-27141-7)
Supplement: Supplementary file 3 — Reporting Summary [file 41467_2021_27141_MOESM3_ESM.pdf]

nature portfolio

Corresponding author(s): Bingzhong Xue

Last updated by author(s): 10/11/2021

Reporting Summary

Nature Portfolio wishes to improve the reproducibility of the work that we publish. This form provides structure for consistency and transparency in reporting. For further information on Nature Portfolio policies, see our Editorial Policies and the Editorial Policy Checklist.

Statistics

For all statistical analyses, confirm that the following items are present in the figure legend, table legend, main text, or Methods section.

n/a	Confirmed	
☐	☒	The exact sample size (*n*) for each experimental group/condition, given as a discrete number and unit of measurement
☐	☒	A statement on whether measurements were taken from distinct samples or whether the same sample was measured repeatedly
☐	☒	The statistical test(s) used AND whether they are one- or two-sided<br>*Only common tests should be described solely by name; describe more complex techniques in the Methods section.*
☒	☐	A description of all covariates tested
☐	☒	A description of any assumptions or corrections, such as tests of normality and adjustment for multiple comparisons
☐	☒	A full description of the statistical parameters including central tendency (e.g. means) or other basic estimates (e.g. regression coefficient) AND variation (e.g. standard deviation) or associated estimates of uncertainty (e.g. confidence intervals)
☐	☒	For null hypothesis testing, the test statistic (e.g. *F*, *t*, *r*) with confidence intervals, effect sizes, degrees of freedom and *P* value noted<br>*Give P values as exact values whenever suitable.*
☒	☐	For Bayesian analysis, information on the choice of priors and Markov chain Monte Carlo settings
☒	☐	For hierarchical and complex designs, identification of the appropriate level for tests and full reporting of outcomes
☒	☐	Estimates of effect sizes (e.g. Cohen's *d*, Pearson's *r*), indicating how they were calculated

Our web collection on statistics for biologists contains articles on many of the points above.

Software and code

Policy information about availability of computer code

Data collection	1. The expression of genes of interest was measured by a one-step quantitative RT-PCR with TaqMan Universal PCR Master Mix reagents (ThermoFisher Scientific, Waltham, MA) using an Applied Biosystems QuantStudio 3 real-time PCR system (ThermoFisher Scientific).<br>2. Histological and IHC images were captured using an Olympus DP73 photomicroscope.<br>3. Immunoblotting was visualized using a Li-COR Imager System (Li-COR Biosciences, Lincoln, NE).<br>4. Food intake, energy expenditure and locomotor activity were measured using PhenoMaster metabolic cage systems (TSE Systems, Chesterfield, MO).<br>5. Body composition representing fat and lean mass was analyzed using a Minispec NMR body composition analyzer (Bruker BioSpin Corporation; Billerica, MA).<br>6. Blood glucose level was measured by OneTouch Ultra Glucose meter (LifeScan, Milpitas, CA).<br>7. Cellular oxygen consumption in brown adipocytes was measured using a XF 96 Extracellular Flux Analyzer (Agilent, Santa Clara, CA)
Data analysis	Western blotting quantification was analyzed using Li-COR Image Studio software (v2.1).<br>Histological and IHC images were analyzed using Olympus CellSens (v1.6).<br>Bioconductor (Version 3.0) and methylKit v0.9.6 were used to analyze RRBS data. All reads were mapped to reference sequences (University of California Santa Cruz Mouse Genome Browser Mouse (NCBI37/mm9) Assembly, https://genome.ucsc.edu/cgi-bin/hgGateway?hgsid=1183225321_q2EV6rSrk1oEv6VFl9obRLdY9dMm).<br>All graphs were made with GraphPad Prism (v9.1.2).<br>Statistical analysis was performed using Microsoft Excel software from Microsoft Office Professional Plus 2019 (version 1808, build 10377.20023, Microsoft Corporation, Redmond, WA, USA) and SPSS software (version 16.0, SPSS Inc, Chicago, IL, USA).

For manuscripts utilizing custom algorithms or software that are central to the research but not yet described in published literature, software must be made available to editors and reviewers. We strongly encourage code deposition in a community repository (e.g. GitHub). See the Nature Portfolio guidelines for submitting code & software for further information.

Data

Policy information about availability of data

All manuscripts must include a data availability statement. This statement should provide the following information, where applicable:

- Accession codes, unique identifiers, or web links for publicly available datasets
- A description of any restrictions on data availability
- For clinical datasets or third party data, please ensure that the statement adheres to our policy

The RNAseq, RRBS and ATAC-seq data have been deposited to Gene Expression Omnibus (GEO) database with the accession code GSE175608 (https://www.ncbi.nlm.nih.gov/geo/query/acc.cgi?acc=GSE175608). All data generated in this study are available and reported. Source data files are included with the paper. The uncropped gel images are included in the source data file.

Field-specific reporting

Please select the one below that is the best fit for your research. If you are not sure, read the appropriate sections before making your selection.

☒ Life sciences ☐ Behavioural & social sciences ☐ Ecological, evolutionary & environmental sciences

For a reference copy of the document with all sections, see nature.com/documents/nr-reporting-summary-flat.pdf

Life sciences study design

All studies must disclose on these points even when the disclosure is negative.

Sample size	The experimental sample sizes were estimated based on our previous published data that are sufficient to detect statistical differences among the groups (e.g. Li et al, VOL. 291, NO. 9, pp. 4523–4536, February 26, 2016; Cui et al, Physiol Rep, 4 (10), 2016, e12799).
Data exclusions	No data were excluded for the analyses.
Replication	All experiments were repeated multiple times as indicated in each figure legend.
Randomization	For all experiments, animals with the same genotypes and gender were randomly assigned into control and experimental groups.
Blinding	We applied blinding to immunohistochemistry sample analysis. For other experiments, blinding is not applicable because all groups including animals and cell experiments were treated the same way thus blinding is not necessary.

Reporting for specific materials, systems and methods

We require information from authors about some types of materials, experimental systems and methods used in many studies. Here, indicate whether each material, system or method listed is relevant to your study. If you are not sure if a list item applies to your research, read the appropriate section before selecting a response.

Materials & experimental systems

n/a	Involved in the study	
☐	☒	Antibodies
☐	☒	Eukaryotic cell lines
☒	☐	Palaeontology and archaeology
☐	☒	Animals and other organisms
☒	☐	Human research participants
☒	☐	Clinical data
☒	☐	Dual use research of concern

Methods

n/a	Involved in the study	
☒	☐	ChIP-seq
☒	☐	Flow cytometry
☒	☐	MRI-based neuroimaging

Antibodies

Antibodies used	All antibodies used in this paper were listed in supplemental table 3.The antibody name, manufacturer and catalog number are listed below:<br>DNMT1 Abcam Ab87654<br>DNMT1 Santa Cruz Biotechnology Sc20701 or sc-271729<br>GFP Aves labs GFP-1010<br>UCP1 Abcam ab23841<br>UCP1 Abcam Ab10983<br>KDM6A (UTX) Abcam ab36938

KDM6A (UTX) Bethyl Laboratories A302-374A
HA Cell signaling technology C29F4
H3K27me3 Abcam ab6002
H3K9me3 Abcam ab8898
H3K4me3 Abcam ab8580
acH3K27 Abcam ab4729
acH3K9 Abcam ab4441
acH3K4 Abcam ab176799
mCherry Abcam ab205402
MyHC DSHB MF20
Perilipin Everest biotech EB07728
PRDM16 Sigma SAB2900806
PRDM16 Sigma SAB3500989
FLAG Sigma F3165
E2F-4 Santa Cruz Biotechnology sc-398543
p107 Santa Cruz Biotechnology sc-250
p130 Santa Cruz Biotechnology sc-374521
α-Tubulin Santa Cruz Biotechnology sc-53646
Biotin-SP (long spacer) AffiniPure Donkey Anti-Rabbit IgG (H+L) Jackson ImmunoResearch 711-065-152
Cy™3 AffiniPure Donkey Anti-Rabbit IgG (H+L) Jackson ImmunoResearch 711-165-152
Alexa Fluor® 488 ffiniPure Donkey Anti-Chicken IgY (IgG) (H+L) Jackson ImmunoResearch 703-545-155
Goat anti-Mouse IgG (H+L) Highly Cross-Adsorbed Secondary Antibody, Alexa Fluor 488 Invitrogen A11029
Goat anti-Mouse IgG (H+L) Highly Cross-Adsorbed Secondary Antibody, Alexa Fluor 594 Invitrogen A11032
Goat anti-Mouse IgG (H+L) Highly Cross-Adsorbed Secondary Antibody, Alexa Fluor 680 Invitrogen A21058
Goat anti-Rabbit IgG (H+L) Highly Cross-Adsorbed Secondary Antibody, Alexa Fluor 680 Invitrogen A21109

Validation

These antibodies were used as recommended by the manufacturer and as cited extensively in previous publications. We have also tested different concentrations of each antibody and used the optimized concentration for each antibody. We further ensured that signals in Western Blots were present at the expected size for each target. The relevant information for each primary and secondary antibodies from the manufacturer and online databases, is as follows:

DNMT1 antibody for Western blotting: Abcam Ab87654, Rabbit polyclonal to Dnmt1. Tested by the manufacturer to detect DNMT1 in western blotting in mouse and human tissue at the expected molecular weight position (183kDa). Seventeen citations from the CiteAb database https://www.citeab.com/antibodies/761843-ab87654-anti-dnmt1-antibody?des=55d3542e7c88c241.

DNMT1 antibody, Santa Cruz Biotechnology Sc20701 or sc-271729 for ChIP assays. Dnmt1 Antibody (H-300) (sc20701) is a rabbit polyclonal antibody, and anti-Dnmt1 Antibody (H-12) (sc271729) is a mouse monoclonal antibody. Both are recommended by the manufacturer for detection of DNMT1 of mouse, rat and human origin by WB, IP, IF, IHC(P) and ELISA. Thirty-four citations for the H-12 antibody from the CiteAb database https://www.citeab.com/antibodies/786245-sc-271729-anti-dnmt1-antibody-h-12?des=9eecfb0027be31e9, and 40 citations for the H-300 antibody from Scbt website: https://www.scbt.com/p/dnmt1-antibody-h-300.

GFP antibody, aves labs GFP1010, Chicken polyclonal antibody. Recommended by the manufacturer to detect GFP by ELISA, ICC, IHC, WB. From manufacturer's notes: Antibodies were analyzed by western blot analysis (1:5000 dilution) and immunohistochemistry (1:500 dilution) using transgenic mice expressing the GFP gene product. One hundred and seventy-three citations from CiteAb database (https://www.citeab.com/antibodies/575207-gfp-1010-green-fluorescent-protein-1-0-mg?des=708459386bbbe89e).

UCP1 antibody for western ABCAM ab23841, Rabbit polyclonal to UCP1. Tested by the manufacturer for detecting UCP1 in mouse, rat and dog by western blotting (WB) and immunohistochemistryPolyclonal Goat IgG (IHC), using mouse and rat brown adipose tissue as positive controls in WB. One hundred and fifteen Citations from the CiteAb database (https://www.citeab.com/antibodies/758073-ab23841-anti-ucp1-antibody?des=641734b5b4769e80).

UCP1 antibody for IHC, ABCAM ab10983, Rabbit polyclonal to UCP1, tested by the manufacturer to detect UCP1 in Mouse, Rat, Spermophilus tridecemlineatus using WB, IHC and immunocytochemistry (ICC). Five hundred and sixty citations from the CiteAb database (https://www.citeab.com/antibodies/758072-ab10983-anti-ucp1-antibody?des=9095b59013736f21).

KDM6A (UTX) antibody, Abcam ab36938, Rabbit polyclonal to KDM6A / UTX. Tested by the manufacturer to detect KDM6A/UTX from mouse, rat and human tissues by western blotting. Thirty citations from the CiteAb database https://www.citeab.com/antibodies/736027-ab36938-anti-kdm6a-utx-antibody?des=a4017169e5665a17.

KDM6A (UTX) antibody, Bethyl Laboratories A302-374A, rabbit polyclonal antibody. Tested by the manufacturer to detect KDM6A/UTX from mouse and human origin by western. Thirty citations from the CiteAb database https://www.citeab.com/antibodies/656618-a302-374a-utx-antibody?des=47c556693cbd3a32.

HA antibody, Cell signaling technology C29F4 rabbit monoclonal antibody #3724. Recommended by the manufacturer to detect HA-tag with WB, IP, IHC, IF and ChIP. One thousand two hundred and five citations from the CiteAb database https://www.citeab.com/antibodies/123379-3724-ha-tag-c29f4-rabbit-mab?des=22e796796cc85c59.

H3K27me3 antibody, Abcam ab6002, ChIP grade mouse monoclonal antibody. Recommended by the manufacturer to detet H3K27me3 in ChIP, ELISA, WB, IHC - Wholemount, ICC/IF from Mouse, Cow, Human origin. Eight hundred and six citations from the CiteAb database https://www.citeab.com/antibodies/763746-ab6002-anti-histone-h3-tri-methyl-k27-antibody-mab?des=594aae60f3098131.

H3K9me3 antibody, Abcam ab8898, ChIP grade rabbit polyclonall antibody. Recommended by the manufacturer to detect H3K9me3

in WB, IHC-P, ICC, ChIP from Mouse, Cow, Human origin. One thousand four hundred and sixty-five citations from the CiteAb database https://www.citeab.com/antibodies/763756-ab8898-anti-histone-h3-tri-methyl-k9-antibody-chi?des=b5387a4b23d8c556.

H3K4me3 antibody, Abcam ab8580, ChIP grade rabbit polyclonall antibody. Recommended by the manufacturer to detect H3K4me3 in ChIP, WB, IHC-P, ICC/IF from Mouse, Cow, Human origin. One thousand seven hundred and ninety-two citations from the CiteAb database https://www.citeab.com/antibodies/763751-ab8580-anti-histone-h3-tri-methyl-k4-antibody-chi?des=0621281dffdd2ade.

acH3K27 antibody, Abcam ab4729, ChIP grade rabbit polyclonall antibody. Recommended by the manufacturer to detect acH3K27 in ChIP, WB, IHC-P, ICC/IF from Mouse, Cow, Human origin. One thousand seven hundred and ten citations from the CiteAb database https://www.citeab.com/antibodies/778149-ab4729-anti-histone-h3-acetyl-k27-antibody-chip-g?des=8da7ecbe20fee13d.

acH3K9 antibody, Abcam ab4441, ChIP grade rabbit polyclonall antibody. Recommended by the manufacturer to detect acH3K9 in ChIP, WB from Mouse, Human origin. Three hundred and seventy-two citations from the CiteAb database https://www.citeab.com/antibodies/778169-ab4441-anti-histone-h3-acetyl-k9-antibody-chip-gr?des=f7ecad4fc26bd028.

acH3K4 antibody, Abcam ab176799, ChIP grade rabbit polyclonall antibody. Recommended by the manufacturer to detect acH3K4 in WB, ICC/IF, Dot blot, ChIP, ChIP-sequencing from Mouse, Human origin. Eleven citations from the CiteAb database https://www.citeab.com/antibodies/2929239-ab176799-anti-histone-h3-acetyl-k4-antibody-epr16?des=fd80e65e6c6a12ec.

mCherry antibody, Abcam ab205402, Chicken polyclonal to mCherry, tested by he manufacturer to detect mCherry in WB, ICC/IF applications. Fifty-two citations from the CiteAb database https://www.citeab.com/antibodies/2929722-ab205402-anti-mcherry-antibody?des=e9c7859d2af9c2f3.

MyHC antibody, DSHB MF20, mouse monoclonal antibody to detect myosin heavy chain (MyHC) protein, tested by the manufacturer to detect MyHC protein in ELISA, FACS, FFPE, Immunofluorescence, Immunohistochemistry, Immunoprecipitation, Western Blot applications from Mouse, Amphibian, Avian, Chicken, Fish, Human, Lizard, Mammal, Pig, Snake, Xenopus, Zebrafish origin. One hundred and sixty-six citations from the manufacturer's website: https://dshb.biology.uiowa.edu/MF-20.

Perilipin antibody, Everest biotech EB07728, goat polyclonal antibody against perilipin-1, reported by the manufacturer and stated in the manufacturer website to detect perilipin in WB, IHC and IF applications in human and mouse tissues. Three citations from the CiteAb database https://www.citeab.com/antibodies/2929722-ab205402-anti-mcherry-antibody?des=e9c7859d2af9c2f3 as well as the manufacturer's website: https://everestbiotech.com/product/goat-anti-perilipin-c-terminus-antibody/.

PRDM16 antibody, Sigma SAB2900806, rabbit polyclonal antibody against PRDM16, reported by the manufacturer to detect PRDM16 in WB, IHC applications in human, rat and mouse tissues.

PRDM16 antibody, Sigma SAB3500989, rabbit polyclonal antibody against PRDM16, reported by the manufacturer to detect PRDM16 in WB, IHC , IF and ELISA applications in human and mouse tissues. Four citations from CiteAb database, https://www.citeab.com/antibodies/2303922-sab3500989-anti-prdm16-antibody-produced-in-rabbit?des=98374910e281a986.

FLAG antibody, Sigma F3165, mouse monoclonal antibody against FLAG, clone M2, reported by the manufacturer to detect FLAG-tag in immunoblotting, immunoprecipitation, immunocytochemistry, immunofluorescence, ELISA, EIA, chromatin immunoprecipitation, electron microscopy, flow cytometry and supershift applications. Seven thousand seven hundred and sixty-four citations from CiteAb database, https://www.citeab.com/antibodies/575237-f3165-monoclonal-anti-flag-r-m2-antibody-produced-in?des=bbed84b4abb81ff8.

E2F-4 antibody, Santa Cruz Biotechnology sc-398543, mouse monoclonal antibody against E2F4, clone D-7, recommended by the manufacturer to detect E2F4 in detection of E2F-4 of mouse, rat and human origin by WB, IP, IF, IHC(P) and ELISA applications. Four citations from CiteAb database, https://www.citeab.com/antibodies/2280468-sc-398543-anti-e2f-4-antibody-d-7?des=c98c4f7a979219a6.

p107/RBL1 antibody, Santa Cruz Biotechnology sc-250, mouse monoclonal antibody against p107/RBL1 protein, clone SD9, recommended by the manufacturer to detect p107/RBL1 of mouse, rat and human origin by WB, IP and IF. Seventy-three citations from CiteAb database, https://www.citeab.com/antibodies/816650-sc-250-anti-p107-antibody-sd9?des=5acef966936f1d4a.

p130/RBL2 antibody, Santa Cruz Biotechnology sc-374521, mouse monoclonal antibody against p130/RBL2 protein, clone A-10, recommended by the manufacturer to detect p130/RBL2 of mouse, rat and human origin by WB, IP, IF and ELISA. Six citations from CiteAb database, https://www.citeab.com/antibodies/816699-sc-374521-anti-p130-antibody-a-10?des=0413ad2602acacf9.

α-tubulin antibody, Santa Cruz Biotechnology sc-53646, mouse monoclonal antibody, recommended by the manufacturer for detection of α Tubulin of broad species origin by WB, IP, IF and ELISA. 127 citations from CiteAb database, https://www.citeab.com/antibodies/835056-sc-53646-anti-tubulin-antibody-10d8?des=43c45b51cd9f31b7.

Cy™3 AffiniPure Donkey Anti-Rabbit IgG (H+L) Jackson Immuoresearch 711-165-152, from the manufacturer website, this secondary antibody has minimal cross reactivity with Bovine, Chicken, Goat, Guinea Pig, Syrian Hamster, Horse, Human, Mouse, Rat, Sheep Serum Proteins. 1132 citations from CiteAb database: https://www.citeab.com/antibodies/2036162-711-165-152-cy3-affinipure-donkey-anti-rabbit-igg-h?des=fe83f7d682eb735d.

Biotin-SP (long spacer) AffiniPure Donkey Anti-Rabbit IgG (H+L) Jackson ImmunoResearch 711-065-152. From the manufacturer website, this secondary antibody has minimal cross reactivity with Bovine, Chicken, Goat, Guinea Pig, Syrian Hamster, Horse, Human, Mouse, Rat, Sheep Serum Proteins. 426 citations from CiteAb database: https://www.citeab.com/antibodies/2035909-711-065-152-biotin-sp-affinipure-donkey-anti-rabbit?des=c6f3624bf1ddfc0d.

Alexa Fluor® 488 AffiniPure Donkey Anti-Chicken IgY (IgG) (H+L) Jackson Immuoresearch 703-545-155, from the manufacturer website, this secondary antibody has minimal cross reactivity with Bovine, Goat, Guinea Pig, Syrian Hamster, Horse, Human, Mouse,

Rabbit, Rat, Sheep Serum Proteins. 462 citations from CiteAb database: https://www.citeab.com/antibodies/2034669-703-545-155-alexa-fluor-488-affinipure-donkey-anti-c?des=2082151ff10573fa.

Alexa Fluor 680 Goat Anti-Rabbit IgG (H / L), highly cross-adsorbed, Invitrogene A21109. According to manufacturer, to minimize cross-reactivity, these goat anti-rabbit IgG (H+L) whole secondary antibodies have been affinity purified and cross-adsorbed against bovine IgG, goat IgG, mouse IgG, rat IgG, and human IgG. Cross-adsorption or pre-adsorption is a purification step to increase specificity of the antibody resulting in higher sensitivity and less background staining. The secondary antibody solution is passed through a column matrix containing immobilized serum proteins from potentially cross-reactive species. Only the nonspecific-binding secondary antibodies are captured in the column, and the highly specific secondaries flow through. The benefits of this extra step are apparent in multiplexing/multicolor-staining experiments (e.g., flow cytometry) where there is potential cross-reactivity with other primary antibodies or in tissue/cell fluorescent staining experiments where there are may be the presence of endogenous immunoglobulins. 225 citations from CiteAb: https://www.citeab.com/antibodies/2401223-a-21109-goat-anti-rabbit-igg-h-l-highly-cross-adso?des=cb3719bfcb178f19.

Goat anti-Mouse IgG (H+L) Highly Cross-Adsorbed Secondary Antibody, Alexa Fluor 488 Invitrogen A11029. From the manufacturer's website, To minimize cross-reactivity, the goat anti-mouse IgG whole antibodies have been highly cross-adsorbed against bovine IgG, goat IgG, rabbit IgG, rat IgG, human IgG, and human serum. Cross-adsorption or pre-adsorption is a purification step to increase specificity of the antibody resulting in higher sensitivity and less background staining. The secondary antibody solution is passed through a column matrix containing immobilized serum proteins from potentially cross-reactive species. Only the nonspecific-binding secondary antibodies are captured in the column, and the highly specific secondaries flow through. Further passages through additional columns result in 'highly cross-adsorbed' preparations of secondary antibody. The benefits of these extra steps are apparent in multiplexing/multicolor-staining experiments where there is potential cross-reactivity with other primary antibodies or in tissue/cell fluorescent staining experiments where there may be the presence of endogenous immunoglobulins. 3308 citations from CiteAb database, https://www.citeab.com/antibodies/2401117-a-11029-goat-anti-mouse-igg-h-l-highly-cross-adsor?des=7ea84c11b75a5e6c.

Goat anti-Mouse IgG (H+L) Highly Cross-Adsorbed Secondary Antibody, Alexa Fluor 594 Invitrogen A11032. From the manufacturer's website, To minimize cross-reactivity, the goat anti-mouse IgG whole antibodies have been highly cross-adsorbed against bovine IgG, goat IgG, rabbit IgG, rat IgG, human IgG, and human serum. Cross-adsorption or pre-adsorption is a purification step to increase specificity of the antibody resulting in higher sensitivity and less background staining. The secondary antibody solution is passed through a column matrix containing immobilized serum proteins from potentially cross-reactive species. Only the nonspecific-binding secondary antibodies are captured in the column, and the highly specific secondaries flow through. Further passages through additional columns result in 'highly cross-adsorbed' preparations of secondary antibody. The benefits of these extra steps are apparent in multiplexing/multicolor-staining experiments where there is potential cross-reactivity with other primary antibodies or in tissue/cell fluorescent staining experiments where there may be the presence of endogenous immunoglobulins. 1157 citations from CiteAb database, https://www.citeab.com/antibodies/2401120-a-11032-goat-anti-mouse-igg-h-l-highly-cross-adsor?des=cb1b2a17ff8a87a2.

Goat anti-Mouse IgG (H+L) Highly Cross-Adsorbed Secondary Antibody, Alexa Fluor 680 Invitrogen A21058. From the manufacturer's website, This secondary antibody is designed for fluorescent Western blot detection on various near-infrared fluorescence instruments. This antibody can be used for multi-color and multiplexing detection when using other antibodies conjugated to compatible Alexa Fluor™ dyes and wavelengths. Other applications of this antibody include immunofluorescent and fluorescent imaging applications when using instrumentation with appropriate excitation and detection capabilities. 190 citations from CiteAb database, https://www.citeab.com/antibodies/2401189-a-21058-goat-anti-mouse-igg-h-l-highly-cross-adsor?des=16aec45644ca7e87

Eukaryotic cell lines

Policy information about cell lines

Cell line source(s)	BAT1 brown adipocyte cell line was provided by Dr. Patrick Seale at University of Pennsylvania. HEK293T cell line was obtained from ATCC.
Authentication	Authentication of BAT1 brown adipocytes was performed by PCR analysis using mouse-specific primers in our laboratory. Authentication of HEK293T cells was done by ATCC with STR profiling (CSF1PO: 11,12; D13S317: 12,14; D16S539: 9,13; D5S818: 8,9; D7S820: 11; TH01: 7, 9.3; TPOX: 11; vWA: 16,19; Amelogenin: X).
Mycoplasma contamination	The cell lines were negative for mycoplasma contamination.
Commonly misidentified lines (See ICLAC register)	No commonly misidentified lines were used in this study.

Animals and other organisms

Policy information about studies involving animals; ARRIVE guidelines recommended for reporting animal research

Laboratory animals	Male and female mice, up to 1 year old, were used in this study. C57BL/6J (Jackson Laboratory #000664), ob/ob (Jackson Laboratory #000632); UCP1-Cre (Jackson Laboratory, Stock No. 024670), UTX-floxed mice (Jackson Laboratory, stock No. 021926), DNMT1-floxed mice (Mutant Mouse Regional Resource Centers (MMRRC, No. 014114), DNMT3a-floxed mice (MMRRC No. 029885), Myf5-Cre (Jackson Laboratory, Stock No. 007893),

	R26-stop-EYFP(Jackson Laboratory #006148).<br>Mice were housed in a temperature- and humidity-controlled animal facility with a 12/12 h light–dark cycle and had free access to water and food (ambient temperature: 20-22°C, humidity: 30-70%).
Wild animals	No wild animals were used in this study.
Field-collected samples	No field-collected samples were used in this study.
Ethics oversight	All animal procedures were approved by the Institutional Animal Care and Use Committee of Georgia State University.

Note that full information on the approval of the study protocol must also be provided in the manuscript.
